# Harnessing the nonthermal effects of ultrasound for cancer treatment: From mechanisms to applications

**DOI:** 10.1002/smo2.70098

**Published:** 2026-08-18

**Authors:** Liu Wang, Huan Tang, Yue Pan, Mingle Li, Weijie Zhao, Kai Liu

**Affiliations:** ^1^ Faculty of Medicine Dalian University of Technology Dalian Liaoning China; ^2^ College of Materials Science and Engineering Shenzhen University Shenzhen China; ^3^ State Key Laboratory of Fine Chemicals Department of Pharmacy School of Chemical Engineering Dalian University of Technology Dalian China; ^4^ Department of Chemistry Engineering Research Center of Advanced Rare Earth Materials (Ministry of Education) Tsinghua University Beijing China

**Keywords:** cancer therapies, cavitation, immunomodulation, mechanical forces, non‐thermal ultrasound

## Abstract

Ultrasound (US) was once predominantly used for diagnosis but not anymore. It has the capability to noninvasively deliver energy to the deep tissues and also enable spatiotemporal control. Unlike high‐intensity focused ultrasound, which primarily ablates tumors through localized heat, nonthermal US converts acoustic energy into mechanical, cavitation, and physicochemical effects. The bioeffects that are induced under nonthermal US provide a mechanistic basis for cancer therapies. Here, we present a mechanism‐guided framework that connects acoustic parameters and nonthermal bioeffects with therapeutic modalities. Nonthermal US‐based cancer treatments are classified into six representative modalities: histotripsy, ultrasound‐targeted microbubble destruction, sonogenetics, sonomechanical therapy (SMT), sonodynamic therapy (SDT), and sonopiezoelectric therapy (SPT). Through a review of recent advances in these modalities, we highlight emerging opportunities and future directions with a focus on standardizing acoustic dosimetry, biosafety assessment, and material optimization. These insights collectively establish a foundation for creating more reliable and clinically relevant nonthermal US‐based therapies for precision oncology.

## Graphical abstract

Nonthermal ultrasound transforms acoustic energy into diverse therapeutic bioeffects through cavitation, mechanical stimulation, and physicochemical activation. This review discusses how acoustic parameters, biological mechanisms, and responsive materials interact to influence nonthermal ultrasound‐enabled cancer therapies. Mechanism‐guided engineering and interdisciplinary integration accelerate the development of precise, effective, and clinically translatable sono‐therapeutic platforms.

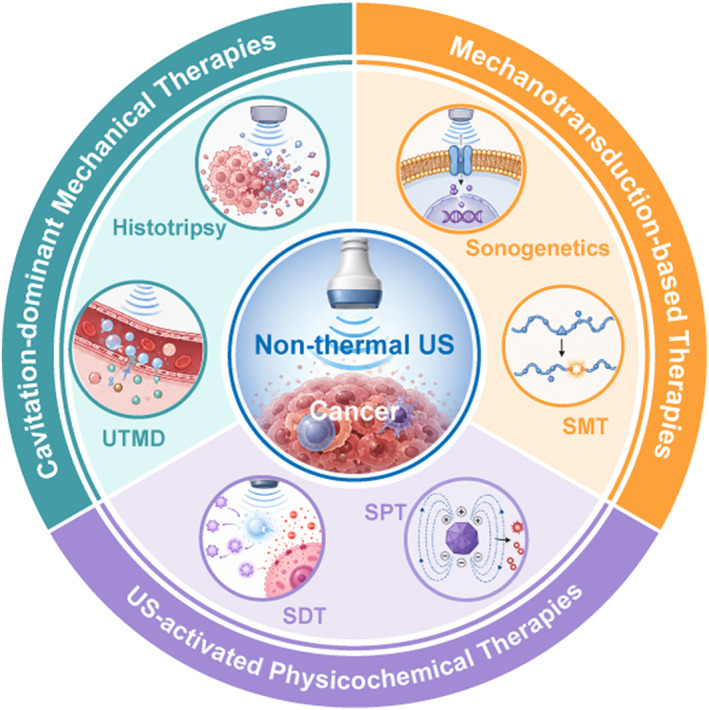

## 1 INTRODUCTION

Over the past few decades, ultrasound (US) technology used as a diagnostic imaging tool has advanced into a wider platform for various therapies.[^1^, ^2^, ^3^] This profound paradigm shift has catalyzed the development of modern ultrasonic medicine. Owing to the physical properties of US, it can deliver acoustic energy to the deep tissues with non‐invasive and spatiotemporal control. The direct effect of therapeutic US on the cellular microenvironment, inducing a specific bioeffect, has led to the development of many novel non‐invasive cancer therapies.

Therapeutic US can be broadly classified into thermal and non‐thermal modalities according to its predominant mechanism of action. High‐intensity focused ultrasound emits acoustic energy at a focal point, rapidly raising temperatures to 80–100°C rapidly. This causes immediate coagulative necrosis of the targeted tissues.[^4^, ^5^] While thermal HIFU is strong enough to destroy localized cancers and prevent their growth or spread, it is not without limitations. In particular clinical situations, anatomical and physiological constraints, such as adjacent critical structures or active blood perfusion, can hinder complete ablation of the tumor.[^6^, ^7^] Additionally, thermal ablation is generally irreversible and often necessitates prolonged treatment times for large tumours. In contrast, several newer treatment strategies involve reversible and precisely controlled biological modulation rather than tissue destruction. As a result, the recent basic and translational research studies have shown interest in using the non‐thermal bioeffects of US in cancer therapies.

Non‐thermal US therapies use various bioeffects to directly kill tumor cells, enhance drug and gene delivery, activate mechanosensitive signaling pathways, and reshape the tumor immune microenvironment, while sparing nearby healthy tissues.[^8^, ^9^] These biological responses mainly result from two related mechanisms: acoustic cavitation and mechanical stimulation.^[10]^ During cavitation, the oscillation and inertial collapse of gas bubbles transiently increase membrane permeability through sonoporation while simultaneously generate sonochemical reactive oxygen species (ROS) via gas‐liquid interfacial reactions. In parallel, acoustic radiation force (ARF) and microstreaming exert mechanical stresses on cells, leading to cytoskeletal remodeling, membrane deformation, and activation of mechanosensitive ion channels.[^11^, ^12^] Because the magnitude and nature of these bioeffects are highly dependent on acoustic parameters and the local biological environment, their therapeutic application requires a mechanistic understanding of how ultrasound exposure governs biological responses and treatment outcomes.

In this review, we establish a mechanism‐oriented framework linking acoustic parameters with non‐thermal bioeffects and therapeutic modalities. The physical parameters governing therapeutic US are first summarized, followed by a discussion of how they regulate key non‐thermal bioeffects. Based on the pathways through which acoustic energy is converted into biological activity, non‐thermal US‐enabled cancer therapies are classified into three categories: cavitation‐dominant mechanical therapies, mechanotransduction‐based therapies, and US‐activated physicochemical therapies. Although these modalities act through distinct mechanisms, they often converge on immune modulation. Accordingly, sono‐immunomodulation is considered a cross‐cutting biological consequence and therapeutic opportunity. By integrating these mechanistically distinct yet biologically interconnected therapeutic strategies, this framework is intended to guide acoustic‐parameter selection, responsive‐material engineering, and the development of clinically translatable non‐thermal US therapies.

## 2 PHYSICAL FOUNDATIONS OF NON‐THERMAL ULTRASOUND

### 2.1 Focused versus non‐focused transducer

**FIGURE 1:**
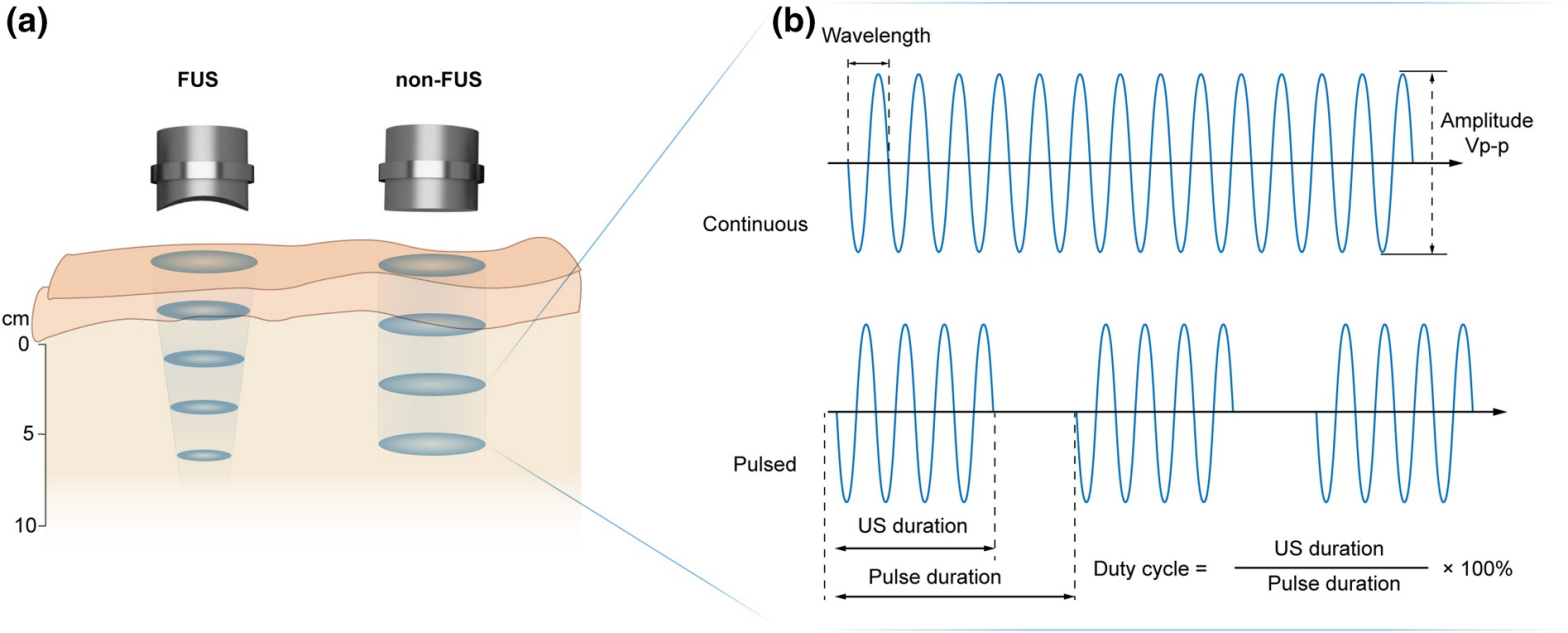
Ultrasound (a) transducers and (b) parameters.

Transducer geometry determines the spatial distribution of acoustic energy and is therefore central to effective sono‐therapies. Focused ultrasound (FUS) employs a specially designed concave transducer to concentrate acoustic energy within a highly localized focal region with high energy density (Figure 1a). Compared with non‐focused ultrasound (non‐FUS), this configuration minimizes the loss of energy to neighbouring tumor‐free tissues and improves deep‐tissue penetration. Therefore, therapeutic effects can be achieved with shorter exposure times and less acoustic energy with FUS.^[13]^ On the other hand, non‐FUS tends to distribute energy more evenly over a larger area of the targeted tissue, making it better for sensitive tissues or repeated exposures. The transducer type affects how acoustic energy is spread, whereas biological effects rely on parameters such as intensity, frequency, and duty cycle (DC).

### 2.2 Frequency

Ultrasonic frequency, defined as the rate of acoustic oscillation, is a pivotal determinant of the functional applications of ultrasound technologies. High‐frequency ultrasound (3–20 MHz) and medium‐frequency ultrasound (0.7–3 MHz) are widely used in diagnostic imaging and therapeutic applications, whereas low‐frequency ultrasound (20–200 kHz) is primarily employed in industrial processes such as cleaning, emulsification, and sonochemistry.^[14]^ From a physical perspective, higher frequencies improve spatial resolution but reduce penetration depth due to increased acoustic attenuation.^[15]^ Energy absorption can cause significant heating, increasing the risk of damage to nearby tissues. Therefore, in vivo therapies typically use frequencies below 1 MHz to strike a good balance between deep tissue penetration and safety.

### 2.3 Duty cycle

The emission mode, whether continuous or pulsed, strongly influences the therapeutic mechanism. Continuous‐wave ultrasound provides a steady stream of acoustic energy at a consistent frequency and power, ideal for treatments that require continuous energy delivery, such as thermal ablation. On the other hand, pulsed‐wave ultrasound delivers energy at intervals. Under pulsed conditions, the DC indicates the proportion of time ultrasound is emitted (Figure 1b). A higher DC results in greater cumulative energy transfer over time, while a lower DC can promote mechanical effects and reduce heat buildup, which is beneficial for modalities like histotripsy and SMT.

### 2.4 Intensity

For therapeutic applications, ultrasound is generally classified into high‐ (100–10000 W/cm^2^), medium‐ (3–100 W/cm^2^), and low‐intensity (<3 W/cm^2^) categories.[^3^, ^16^] Ultrasound output is often characterized by two key metrics: spatial‐peak pulse‐average intensity (I_SPPA_) and spatial‐peak temporal‐average intensity (I_SPTA_). I_SPPA_ represents the maximum energy delivered per pulse, vital for triggering transient bioeffects such as cavitation and membrane sonoporation. In contrast, I_SPTA_ indicates the average energy over time, which is especially relevant for long‐term exposure. In a US‐related study, the relationship between I_SPTA_ and I_SPPA_ is given by Equation (1).^[17]^

(1) $$I_{\text{SPTA}}=I_{\text{SPPA}}\times \text{Duty}\;\text{cycle}$$

Acoustic pressure is an essential mechanical parameter for characterizing the ultrasonic field and measuring the mechanical forces acting on cells and tissues, unlike intensity, which measures energy flux. This helps regulate non‐thermal bioeffects and ensures result consistency. Excessive acoustic pressure may cause an inadvertent tissue injury, highlighting the importance of accurate pressure measurement. Additionally, relying only on I_SPTA_ for classifying ultrasound exposure may be inaccurate for non‐thermal therapies. Several approaches discussed in this review, particularly histotripsy, use very high peak negative pressures (>10 MPa) and high instantaneous peak intensities, but operate at low duty cycles (<1%).[^18^, ^19^] As a result, their temporal‐average intensities may remain within the low‐intensity range despite strong instantaneous mechanical effects. Throughout this review, the term “non‐thermal therapeutic ultrasound” is used as a broad descriptor for ultrasound modalities that achieve cancer treatment primarily through non‐thermal mechanisms. This definition encompasses modalities spanning a wide range of acoustic output and exposure conditions, provided that their therapeutic effects arise predominantly from mechanical or physicochemical bioeffects, including downstream immunological consequences, rather than from heat‐mediated tissue damage.

## 3 NON‐THERMAL BIOEFFECTS

Non‐thermal US operates through two interconnected physical mechanisms: cavitation‐mediated effects, which arise from the oscillation or collapse of gas bubbles, and non‐cavitational mechanical effects, including ARF and acoustic streaming. Together, these processes convert acoustic energy into coordinated physical, chemical, and biological responses, providing the mechanistic basis for non‐thermal ultrasound‐enabled cancer therapies.

### 3.1 Cavitation effects

**FIGURE 2:**
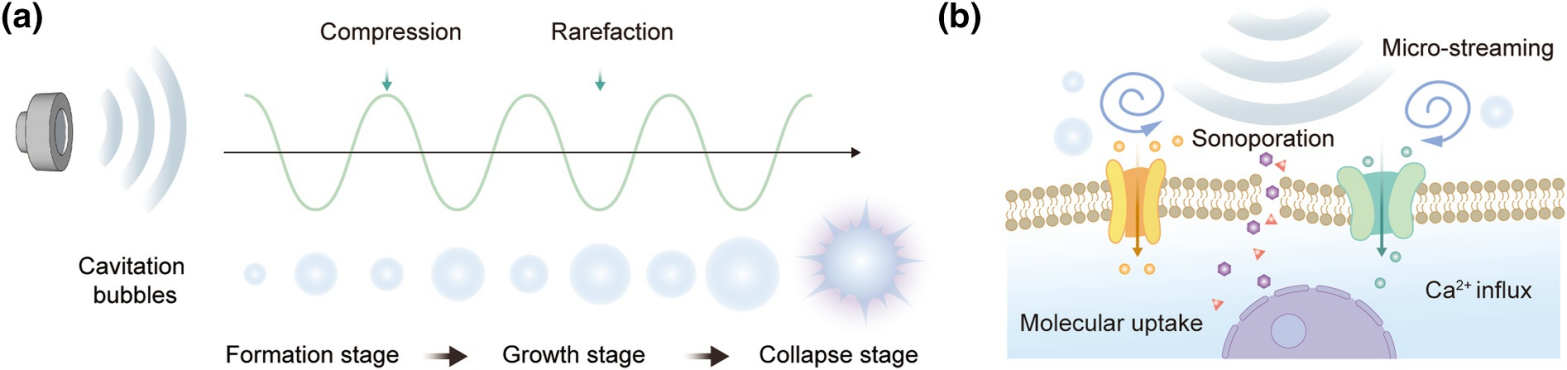
Schematic image of (a) the acoustic cavitation process and (b) the related mechanical forces.

Under alternating acoustic pressure, microbubbles oscillate and expand during the rarefaction phase (Figure 2a).^[20]^ Below the inertial cavitation threshold, stable cavitation involves continuous, nonlinear bubble oscillation that produces steady microstreaming and moderate shear stresses. These mechanical effects support reversible sonoporation, enhance molecular transport, and facilitate drug and gene delivery (Figure 2b). Once the peak negative pressure exceeds the inertial cavitation threshold, bubbles collapse violently and quickly, forming localized hotspots with temporary temperatures around 4000–25000 K and pressures over 80 MPa.^[11]^ These extreme conditions initiate sonochemical reactions, leading to the formation of hydroxyl radicals (·OH) and hydrogen atoms (H·) via molecular dissociation.[^21^, ^22^, ^23^] Therefore, these cavitation‐driven “acoustic reactor” effects form a key mechanistic foundation for US‐enhanced oxidative treatments, such as SDT.

Besides sonochemical ROS production, sonoluminescence caused by inertial cavitation is another important non‐thermal bioeffect. When cavitation bubbles collapse rapidly, they create extreme, localized conditions that produce transient light emissions, typically spanning ultraviolet to visible wavelengths. It acts as an internal excitation source for photosensitizers.^[24]^ This phenomenon provides a mechanistic explanation for sono‐photodynamic synergy, where cavitation‐produced photons help activate photosensitizers and enhance direct sonochemical ROS generation.

In addition to its physicochemical effects, cavitation induces mechanical disturbances, including microstreaming, shear stress, microjets, shock waves, and transient membrane permeabilization. These mechanical forces connect local bubble behavior to subsequent biological effects, such as increased membrane transport, vascular damage, immunogenic cell damage and changes in the tumor microenvironment. Cavitation is a multimodal, non‐thermal bioeffect that combines mechanical disruption, sonochemical ROS generation, and sonoluminescence excitation. The main challenge is controlling these interconnected effects with precise spatial and temporal regulation.

### 3.2 Non‐cavitational mechanical effects

As ultrasound propagates through biological tissues, oscillatory acoustic pressures generate mechanical forces independent of cavitation or exogenous microbubbles.^[25]^ These non‐cavitational mechanical effects include ARF, radiation pressure‐induced tissue deformation, and acoustic streaming, which together impose compressive, tensile, and shear stresses on cells and extracellular matrices.[^26^, ^27^] Unlike cavitation‐mediated microstreaming, which originates from oscillating gas bubbles, these mechanical forces arise directly from momentum transfer during acoustic wave propagation.

These mechanical stimuli are transduced into cellular responses through mechanotransduction pathways. ARF and associated tissue deformation can perturb the plasma membrane, cytoskeleton, focal adhesions, and extracellular matrix, thereby activating mechanosensitive ion channels such as Piezo1/2 and TRPV4. Downstream signaling commonly involves calcium influx, Rho GTPase activity, cytoskeletal remodeling, YAP/TAZ nuclear translocation, and ERK1/2‐ or Wnt/β‐catenin‐associated pathways.[^28^, ^29^, ^30^, ^31^] Together, these responses regulate membrane permeability, cell‐cycle progression, migration, apoptosis sensitivity, and immune‐related signaling.

However, when microbubbles or other cavitation nuclei are present, local mechanical stresses can be substantially amplified through stable cavitation, microstreaming, and bubble collapse. Accordingly, cavitation‐mediated and non‐cavitational mechanical effects should be considered as mechanistically distinct yet functionally complementary pathways through which non‐thermal US regulates tumor cells and the tumor microenvironment.

### 3.3 Acoustic parameter modulation of therapeutic mechanisms

The relative contributions of cavitation and non‐cavitational mechanical effects are determined by the interplay among acoustic pressure, frequency, pulse duration, DC, exposure time, and the presence of cavitation nuclei or ultrasound‐responsive materials. Among these variables, peak rarefactional pressure and ultrasound frequency are the primary determinants of cavitation activity. Their combined influence is commonly described by the mechanical index (MI), defined as the peak rarefactional pressure divided by the square root of the ultrasound frequency.[^32^, ^33^] In general, a higher MI indicates a greater likelihood of cavitation. Lower frequencies cause bubble growth and collapse at lower pressure amplitudes, whereas high frequencies typically require high pressures and are more affected by acoustic attenuation. Additionally, cavitation thresholds can vary significantly based on the propagation medium, dissolved gases, pulse sequences, and existing nuclei. They may also depend on microbubble formulation and acoustic tissue properties. Solutions or substances that are particularly suited for inertial cavitation techniques such as histotripsy, ultrasound‐targeted microbubble destruction (UTMD), and oxidative therapies have been proven to have variable cavitation thresholds. In this way, each biological environment will require specific fine‐tuning of the acoustic exposure parameters, cavitation nuclei and formulation conditions.

Duty cycle and pulse structure influence cavitation bioeffects, whether mechanical disruption or oxidative stress. Pulsed exposure with a low DC produces high peak rarefactional pressure without causing cumulative heating. This tends to enhance mechanical effects, including tissue fractionation in histotripsy or membrane permeabilization in selected UTMD protocols. On the other hand, longer pulses and higher duty cycles amplify the average intensity with time while increasing the bubble activity. The possibility of biological responses to oxidative stress, other than relevant environmental stressors, and the risk of overheating. The DC is an important factor, but not the only factor, determining whether cavitational effects are more favourable towards the disruption of mechanical disruption or oxidative stress.

Mechanical forces not associated with cavitation induce deformation in cells, the extracellular matrix, and tissue matrix. These deformations then trigger the activity of mechanosensitive ion channels, cytoskeletal remodeling, focal adhesion signaling, and various other downstream mechanotransduction pathways. Since these responses can occur without cavitation, they offer a mechanistic foundation for sonogenetics and SMT. However, microbubbles or other cavitation nuclei can significantly increase local mechanical stresses, making it difficult to distinguish between cavitation‐mediated and non‐cavitational mechanisms.

**FIGURE 3:**
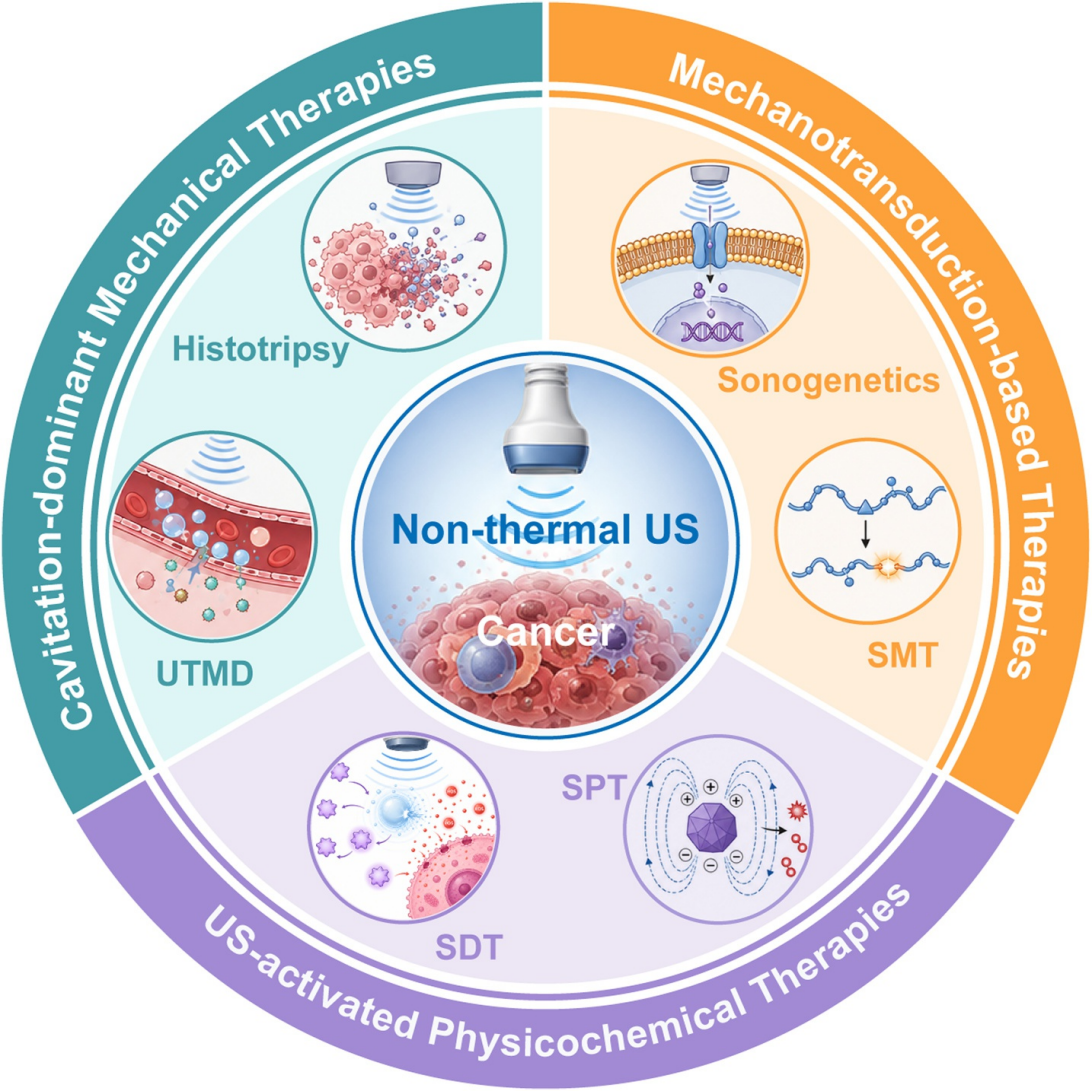
Mechanism‐guided classification of non‐thermal ultrasound‐based cancer therapies according to parameter‐dependent bioeffects.

This multidimensional parameter space offers a mechanistic foundation for the therapeutic approaches outlined below (Figure 3). Table 1 summarizes the key features of main non‐thermal ultrasound therapeutic modalities, highlighting their mechanisms, acoustic features, therapeutic applications, and current translational status. Cavitation‐dominant mechanical therapies, such as histotripsy and UTMD, rely on bubble oscillation or collapse to break down tissue, disrupt blood vessels, and enhance molecular delivery. Mechanotransduction‐based therapies, including SMT and sonogenetics, utilize ultrasound‐induced mechanical stresses to trigger mechanochemical reactions or activate signaling pathways sensitive to mechanical stimuli. US‐activated physicochemical therapies, such as SDT and SPT, use acoustic energy to activate sonosensitizers or piezoelectric materials, thereby generating ROS, charge separation, local electric fields, and biochemical stress responses. Across these therapeutic modalities, immune modulation is a common outcome resulting from tumor cell damage, antigen release, vascular changes, and inflammatory signals.

**TABLE 1:** Comparative analysis of non‐thermal ultrasound‐based cancer therapeutic modalities.

Modality	Core mechanism	Key parameters	Material needs	Status	Advantages	Limitations
Histotripsy^[34]^	Inertial cavitation; tissue fractionation	250 kHz‐5 MHz; 10–30 MPa; duty cycle <2%;	No exogenous agents	FDA‐cleared for liver tumors	Non‐invasive; precise ablation; real‐time imaging; immune activation potential	Limited near ribs/bowel gas; deep or large lesions; long‐term evidence needed
UTMD^[35]^	Stable/inertial cavitation; enhanced vascular and membrane permeability	1–2 MHz; 0.5–3.0 W/cm^2^; most 50% duty cycle	Microbubbles ± therapeutic drug	Early clinical trials	Targeted drug/gene delivery; reversible BBB opening; imaging compatible	Vascular damage at high pressure; parameter standardization lacking
Sonogenetics^[36]^	Ultrasound‐activated mechanosensitive channels	Most 0.5 MHz; 0.5–3.0 W/cm^2^; 0.05–0.5 Mpa; 40%–50% duty cycle	Engineered mechanosensitive protein	Mostly preclinical	Programmable, cell‐specific, spatiotemporal modulation	Gene delivery challenges; regulatory concerns; limited tumor specificity
SMT^[37]^	Force‐induced bond scission and mechanochemical activation	20 kHz‐1 MHz; 1.47 Mpa; 4.5% duty cycle, 3.6 W/cm^2^	Mechanophores or force‐responsive materials	Early preclinical	On‐demand molecular activation; minimal off‐target effects	Limited mechanophore library; low deep‐tissue efficiency; limited translational evidence
SDT^[38]^	Cavitation‐enhanced ROS generation	0.5–3 MHz; <3 W/cm^2^; pulse or continuous US; 10%–100% duty cycle	Sonosensitizers	Early clinical trials	Deep tissue penetration; combinable with chemotherapy, immunotherapy, and nanomedicine	Limited tumor selectivity of current sonosensitizers; dosimetry and standardization needed
SPT^[39]^	Piezoelectric charge separation and ROS generation	Similar acoustic settings to SDT	Piezoelectric nanomaterials	Preclinical	Oxygen‐independent or hypoxia‐tolerant activity	Material biodegradation, long‐term biosafety, and clearance remain concerns

## 4 NON‐THERMAL ULTRASOUND THERAPEUTIC MODALITIES IN ONCOLOGY

### 4.1 Cavitation‐dominant mechanical therapies

Mechanical procedures that utilize cavitation as a primary mechanism are the most direct applications of acoustic cavitation in oncology since they predominantly utilize the mechanical stresses arising from the oscillation and collapse of bubbles. The shock waves and other forces that occur following the explosion either directly disrupt tumor tissue or transiently permeabilize it to allow therapeutic delivery. Two representative modalities refer to this therapy. Using short pulse duration and high peak pressure, histotripsy is capable of producing dense cavitation clouds at precise locations in tissue. This results in mechanical fractionation of tissue into acellular debris. As a result, histotripsy provides a non‐thermal and non‐ionizing ablation method for solid tumours. At lower acoustic pressures, UTMD uses exogenously administered microbubbles as cavitation nuclei, which causes stable and inertial cavitation to enhance vascular and tissue permeability to enable drug and gene delivery. The therapeutic efficacy of both approaches is based on the precise spatiotemporal control of cavitation‐driven mechanical force, despite their differing therapeutic endpoints.

#### 4.1.1 Histotripsy

**FIGURE 4:**
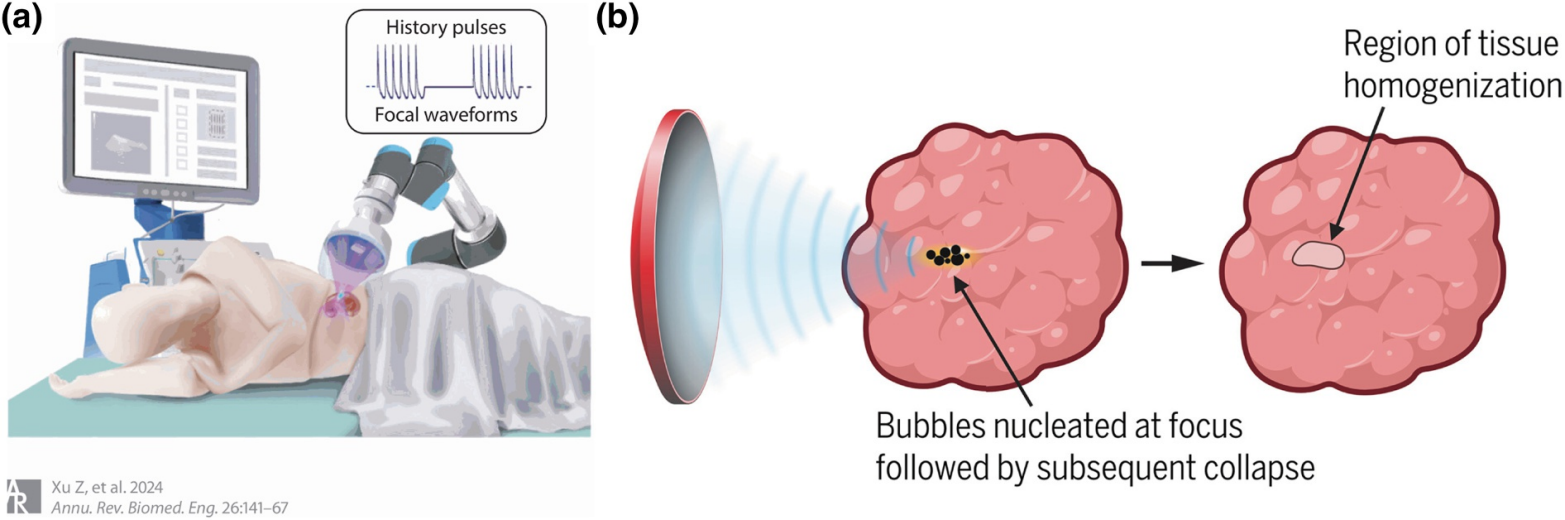
Schematic illustration of histotripsy treatment. (a) A pulsed beam is focused through the skin and translated either mechanically or electronically over the target volume to liquefy tissue. Reproduced under terms of the CC‐BY license.^[18]^ Copyright 2024, The Author(s), published by Annual Reviews. (b) Histotripsy uses high peak rarefactional pressures to nucleate bubbles that rapidly expand and collapse, homogenizing tissue. Reproduced with permission.^[1]^ Copyright 2024, American Association for the Advancement of Science.

Histotripsy is a non‐invasive, non‐ionizing, and non‐thermal ablation modality that is guided by real‐time imaging.^[40]^ This technique employs high‐pressure acoustic pulses with microsecond durations to generate dense cavitation clouds that mechanically fractionate tumor tissue into acellular debris while minimizing thermal damage to adjacent structures (Figure 4). After treatment, the liquefied necrotic tissue in the ablation zone is usually absorbed within 1–2 months, resulting in minimal residual scarring. In addition to destroying tumors locally, histotripsy strongly stimulates the immune response to tumors. The mechanical breakdown of tissue releases tumor‐associated antigens (TAAs), damage‐associated molecular patterns (DAMPs), and pro‐inflammatory cytokines, which help mature dendritic cells and activate cytotoxic T lymphocytes (CTLs).[^41^, ^42^] Several preclinical studies have also shown that histotripsy can generate systemic antitumor responses, including abscopal effects on untreated distant tumors.[^43^, ^44^] These results indicate that histotripsy serves as both a local ablative technique and a potential trigger for systemic cancer immunotherapy.

As an emerging therapeutic modality, histotripsy is being investigated for the treatment of a wide range of solid tumors, including those of the liver, kidney, and brain. Its non‐thermal and non‐invasive characteristics are particularly advantageous for treating lesions located near critical anatomical structures. Notably, histotripsy has received FDA clearance for the treatment of primary liver tumors, including hepatocellular carcinoma (HCC) and neuroendocrine tumors, as well as liver metastases derived from colorectal and breast cancers. The clinical safety and feasibility of histotripsy were highlighted in a 2019 Phase I trial (NCT03741088), which included 8 patients with 11 malignant liver tumors.^[45]^ Two months after treatment, localized regression was observed in all targeted tumors, and no treatment‐related adverse events were reported. Building on these encouraging findings, the first device capable of the technique was cleared by the United States Food and Drug Administration in October 2023 for the destruction of liver tumors. A large multicenter clinical trial (NCT04573881) was initiated in 2021 across Germany, Italy, Spain, and the United Kingdom and remains ongoing with 5 years of follow‐up visits.[^29^, ^30^, ^31^, ^32^] Although clinical outcomes appear promising, there are limitations to histotripsy. The most effective treatment for solid tumors of well‐defined nature in accessible organs such as liver and kidney. Nonetheless, it can not be used for diffuse and infiltrative tumors and those located near visceral vessels. The accuracy of targeting may be influenced by tissue heterogeneity and respiratory motion, necessitating sophisticated systems for image‐guidance. Moreover, the long‐term immunological effects of histotripsy‐induced release of tumor debris and the risk of a systemic autoimmune process are poorly understood. To promote wider clinical adoption of these products, efforts should focus on enhancing the monitoring of treatment responses, developing more effective anti‐targeting algorithms, and conducting comprehensive immunological safety studies.

#### 4.1.2 Ultrasound‐targeted microbubble destruction (UTMD)

Ultrasound‐responsive microbubbles (MBs) play an important role in UTMD. These MBs serve as cavitation nuclei and mechanical amplifiers that convert the acoustic energy present in ultrasound waves into localized biological effects. The selection of the gas core, shell composition, size range and surface functionalization primarily determines their performance.

Clinically approved MBs are generally composed of a high‐molecular‐weight inert gas core, such as perfluorocarbons or sulfur hexafluoride, surrounded by a shell made of phospholipids or proteins.^[46]^ The gas improves circulation time by limiting gas diffusion, while the shell regulates the compressibility, oscillation behaviour and acoustic stability. MBs with lipid shells, and especially those having phospholipid monolayers (e.g., DSPC and DPPC), show large oscillation amplitudes at low acoustic pressures. As a result, they are ideal candidates for non‐thermal US applications.[^47^, ^48^] Polymer‐shelled MBs (e.g., PLGA and PVA) are more mechanically robust; however, they usually need higher acoustic pressures to trigger cavitation. On the other hand, protein‐shelled MBs like albumin‐based MBs offer an improved balance between biocompatibility and shell strength.[^47^, ^49^]

**FIGURE 5:**
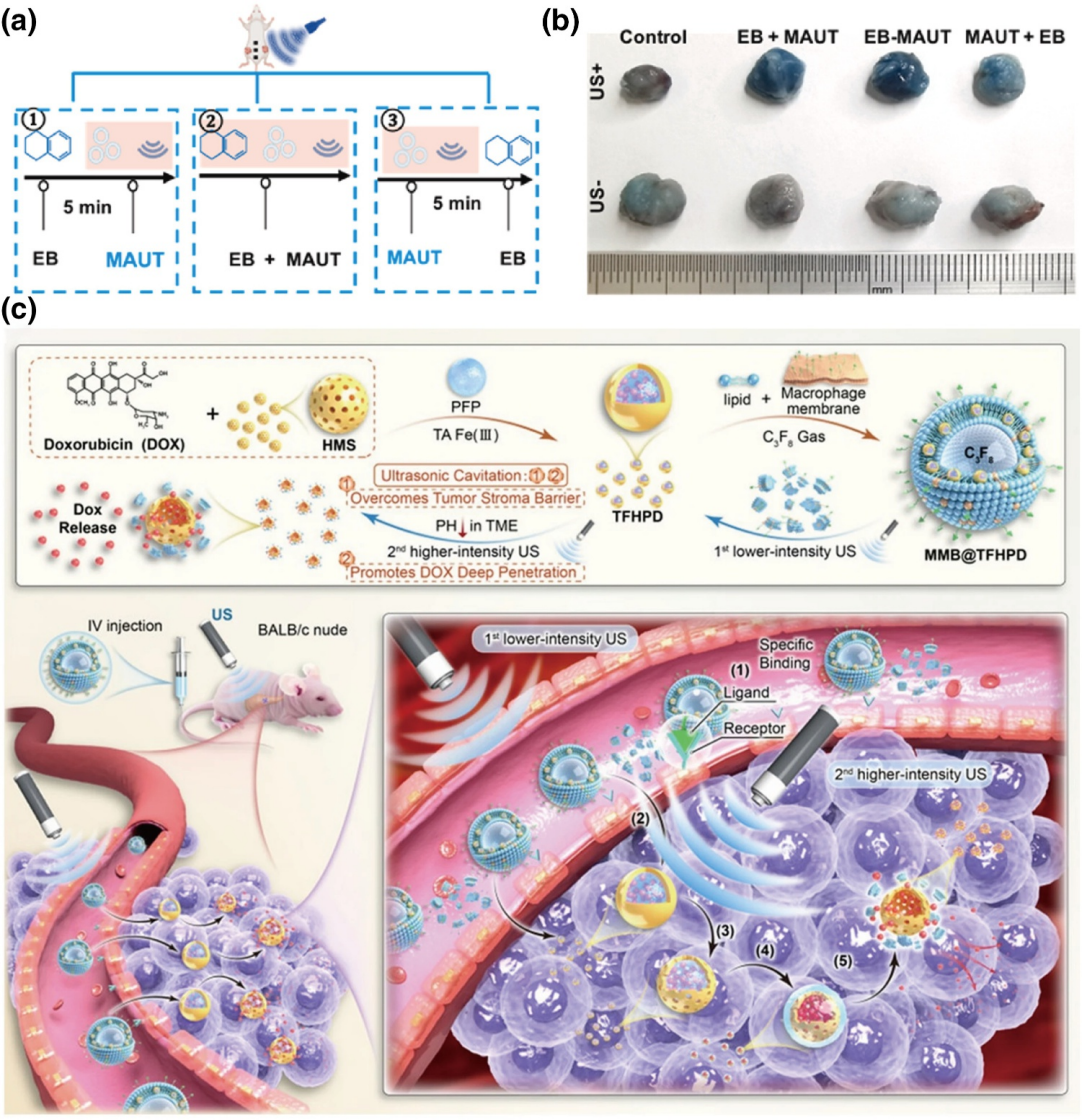
Ultrasound‐targeted microbubble destruction (UTMD) promotes chemical drug delivery. (a) The detailed schematic illustration of MB‐assisted ultrasonication treatment. (b) The digital photos of tumors after different treatments as depicted. The blue color in tumors indicates Evans blue (EB) accumulation. Reproduced with permission.^[50]^ Copyright 2023, Elsevier. (c) Schematic representation of sequential US‐activated MB‐NP hybrid (MMB@TFHPD) for robust tumor suppression in PDAC. Reproduced with permission.^[51]^ Copyright 2025, Wiley‐VCH.

The size of microbubbles is an essential design parameter as it alters the resonance frequency. In general, clinically approved MBs have a diameter in the range of 1–10 μm. This allows for effective oscillation (without rupture of the microbubble) in diagnostic and therapeutic ultrasound while remaining within the vascular system.^[52]^ Stable cavitation is preferred in UTMD as sustained oscillation of microbubbles generates manageable mechanical forces, including acoustic microstreaming, shear stress and reversible sonoporation. The drugs cause a temporary increase in permeability of blood vessels and cells, thus improving delivery to the target tissue ulcer maximally while damage to adjacent healthy tissue is minimized. The therapeutic potential of this approach is indicated by improved penetration of the tracer Evans blue (Figure 5a,b). These findings indicate that UTMD‐mediated cavitation can partially overcome the high interstitial fluid pressure typical of solid tumors, enabling a more effective intratumoral delivery of therapeutic agents.^[50]^

Besides promoting cavitation, MBs serve as therapeutic carriers. Chemotherapeutics, nucleic acids, proteins and nanoparticles can be carried within the shell of the microbubble, conjugated to the microbubble surface or co‐administered for ultrasound‐controlled release. Targeting ligands can help enhance retention in tumor vasculature or specific cell populations for better localized delivery and reduced off‐target effects. Biomimetic engineering has broadened these capabilities. For example, the macrophage membrane‐cloaked TFHPD platform employs a dual‐ultrasound strategy to enhance vascular extravasation and deep intratumoral penetration, thereby improving therapeutic distribution throughout the tumor (Figure 5c).^[51]^ The initial ultrasound pulse induces microbubble cavitation, thereby facilitating vascular extravasation and promoting the accumulation of the nanocarriers within tumor tissue. Subsequent higher‐intensity pulse then triggers acoustic droplet vaporization of the perfluoropentane core. The resulting mechanical forces remodel the tumor extracellular matrix, thereby promoting deep intratumoral drug release and improving therapeutic penetration into the tumor core. This strategy helps overcome the elevated interstitial fluid pressure and dense stromal barriers characteristic of solid tumors.

**FIGURE 6:**
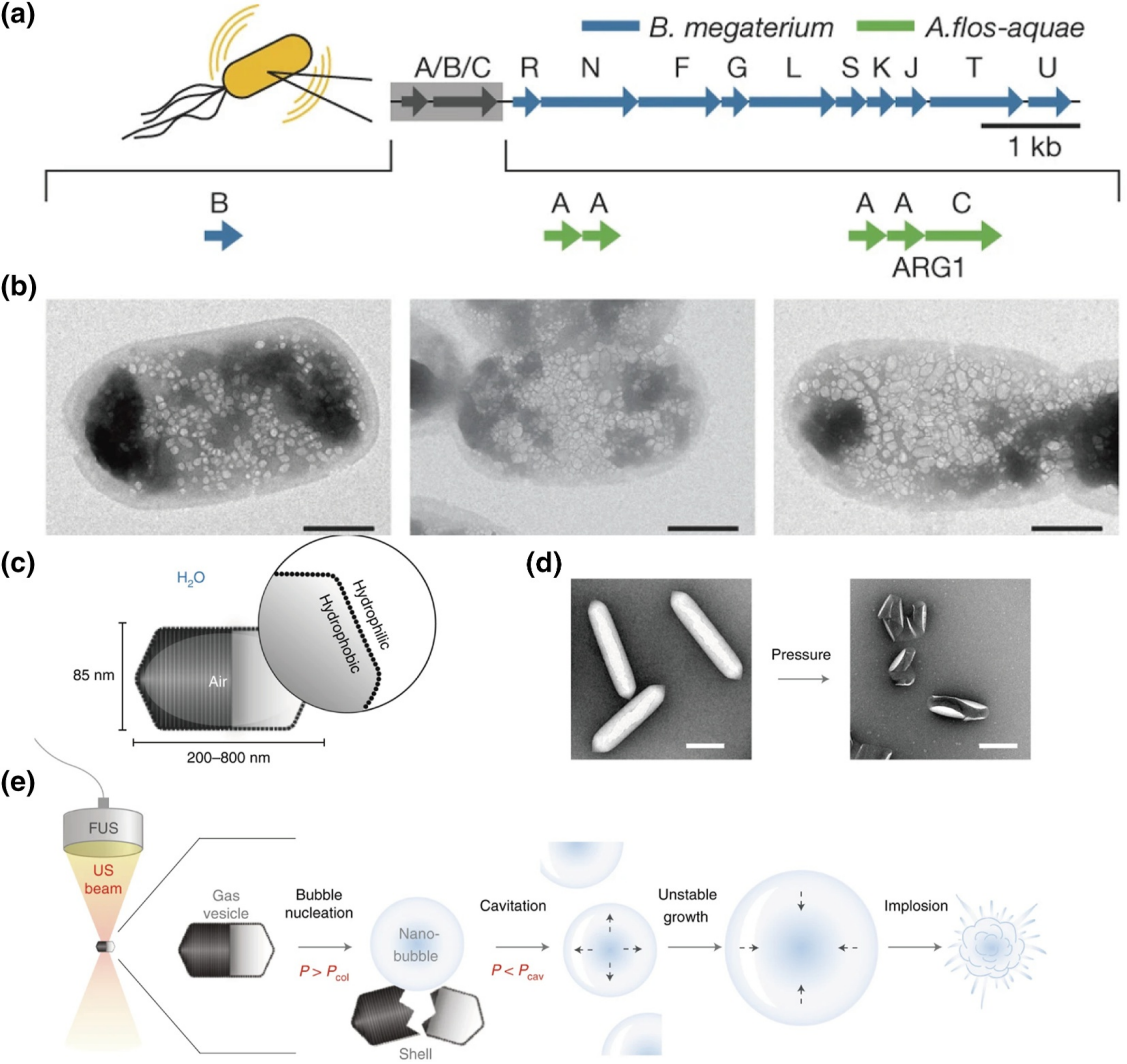
GVs act as seeds for ultrasonic cavitation. (a) Organization of ARG clusters. (b) TEM images of GVs in and isolated from (*Escherichia coli*) Reproduced with permission.^[53]^ Copyright 2018, Springer Nature. (c) Schematic drawing of a GV. The GV's amphiphilic protein shell encloses a stable, gas‐filled structure. (d) Representative transmission electron microscopy images of intact (left) and collapsed (right) Ana GVs. (e) Proposed mechanism of GV‐seeded cavitation. Reproduced with permission.^[54]^ Copyright 2021, Springer Nature. ARG, acoustic reporter gene; GVs, gas vesicles.

Expanding beyond traditional MB systems, Professor Mikhail Shapiro's group pioneered the use of acoustic reporter genes (ARGs) to biosynthesize nanoscale gas vesicles (GVs) with ultrasound‐responsive properties.^[53]^ Expression of these ARGs in *Escherichia coli* produces genetically engineered bacteria (GEBs) that function as programmable biomolecular platforms (Figure 6a,b). GVs are biconical, protein‐shelled nanostructures that self‐assemble by excluding water and sequestering gas from the cytoplasm, forming stable gas‐filled cavities (Figure 6c).^[54]^ Unlike exogenous phase‐transition nanoparticles, these GEBs provide endogenous cavitation nuclei and function as biologically derived agents for ultrasound‐mediated therapy (Figure 6d). In vivo studies in tumor‐bearing mice demonstrated that GEBs were progressively cleared from major organs while preferentially colonizing and proliferating within the tumor microenvironment. Upon exposure to low‐frequency ultrasound, these nanoscopic GVs expand into micrometer‐scale cavitating bubbles that generate localized mechanical forces (Figure 6e). This remotely triggered process facilitates localized cell killing and tissue disruption, while also enabling programmed GEB lysis to release the payload, thereby validating the therapeutic synergy of this genetically engineered platform.

Notably, UTMD also exerts important immunomodulatory effects by promoting immune cell infiltration, increasing the intratumoral accumulation of immunotherapeutic agents, and alleviating extracellular matrix barriers through cavitation‐mediated vascular permeabilization and mechanical disruption.^[55]^ Through these mechanisms, UTMD appears to be an attractive platform for combination immunotherapy including immune checkpoint blockade, cytokine delivery, and adoptive cell therapy. Nonetheless, the treatment efficacy is largely dependent on optimization of both microbubble formulation as well as the acoustic parameters. Models of MBs are often short‐lived and fail to penetrate blood vessels. These limitations restrict the treatment to only well‐vascularized tumors and cancers, such as pancreatic cancer, which are poorly vascularized are poorly treated. Future advances will require the development of next‐generation targeted cavitation agents, standardized acoustic dosimetry, and clinically translatable ultrasound delivery systems.

### 4.2 Mechanotransduction‐based therapies

Mechanotransduction‐based therapies exploit the mechanical forces generated during ultrasound propagation through biological tissues independently of microbubble cavitation. Sonogenetics employs genetic engineering to express mechanosensitive ion channels in target cells, enabling US‐triggered activation of intracellular signaling pathways and selective tumor cell killing through controlled ion flux. In contrast, SMT exploits ultrasonic mechanochemistry to convert US‐generated mechanical forces into molecular transformations, enabling controlled drug release, prodrug activation, and mechanically driven therapeutic functions. Although sonogenetics and SMT operate at different biological scales (cell signaling vs. molecular bond modulation), both convert non‐cavitational mechanical forces into programmable therapeutic outputs.

#### 4.2.1 Sonogenetics

**FIGURE 7:**
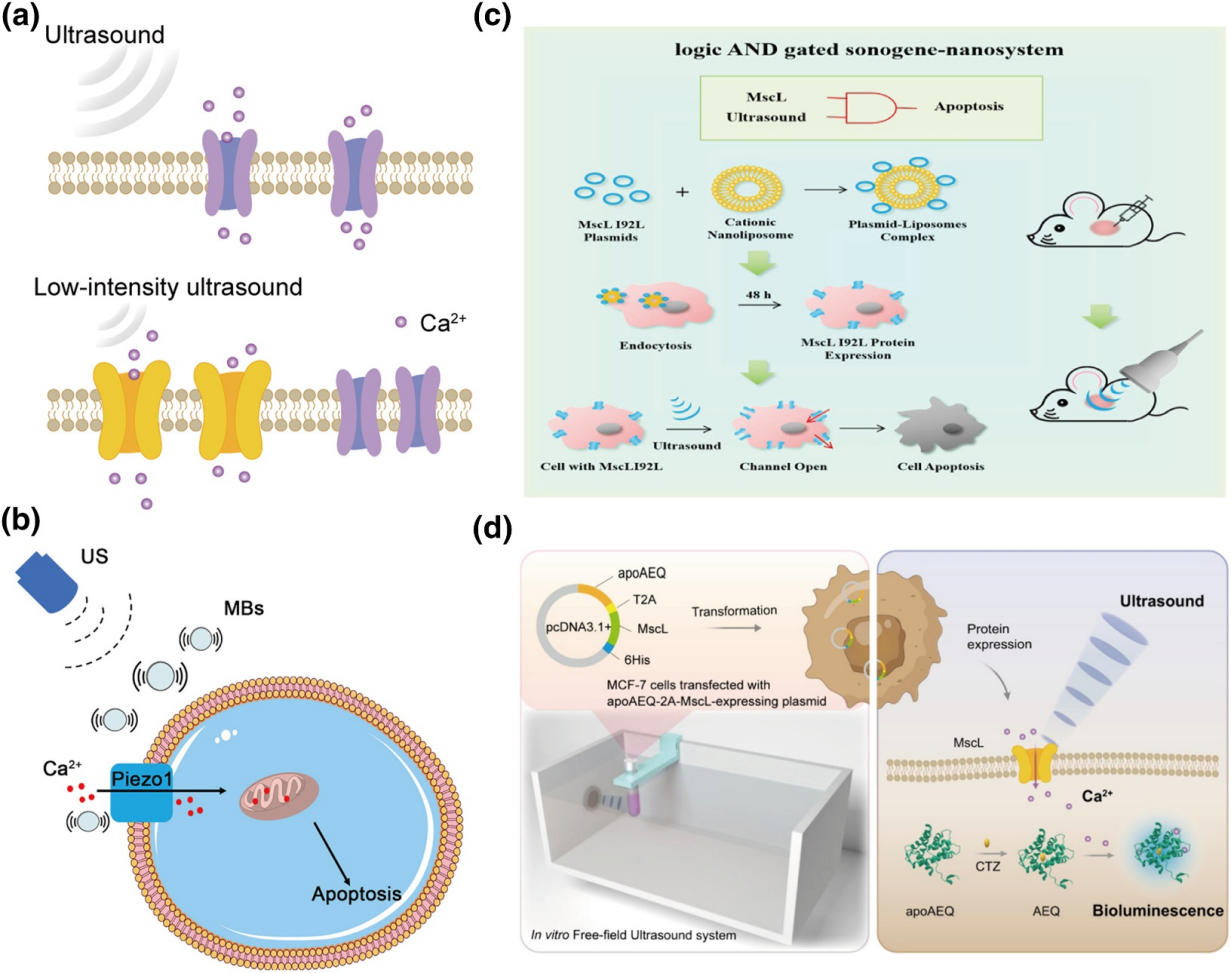
Sonogenetic strategies for cancer treatment. (a) Sonogenetic proteins decrease the celler response threshold to ultrasound. (b) Piezo1 expression in PDAC enables a noninvasive treatment strategy using US and MBs. Reproduced with permission. Reproduced with permission.^[56]^ Copyright 2022, Elsevier. (c) Logic AND‐gated sonogenetics for regulating tumor‐cell apoptosis. Reproduced with permission.^[57]^ Copyright 2020, American Chemical Society. (d) Proposed luminescence mechanism of a mechanoresponsive bioluminescent protein under ultrasound irradiation. Reproduced with permission. Reproduced with permission.^[58]^ Copyright 2025, American Chemical Society.

Sonogenetics, specifically referring to sono‐mechanogenetics in this review, is an emerging field that employs genetic engineering to express mechanosensitive proteins in target cells and has developed as a complementary approach to optogenetics, electrogenetics, and magnetogenetics.^[59]^ Upon ultrasound stimulation, these sonogenetic transducers convert mechanical stimuli from ultrasound into biochemical signals that initiate downstream intracellular signaling cascades. However, endogenous mechanosensitive ion channels can narrow the effective acoustic operating window and introduce substantial background activity. Consequently, improving the sensitivity of sonogenetic proteins is critical for enabling non‐thermal ultrasound stimulation while minimizing nonspecific off‐target effects (Figure 7a). A major challenge in the field remains the identification of optimal ultrasound‐responsive proteins, with current research primarily focused on membrane‐associated mechanosensitive ion channels.^[60]^

The mammalian mechanosensitive ion channels Piezo1 and Piezo2 were first identified in 2010.^[30]^ Piezo1 is a non‐selective cation channel with high Ca^2+^ permeability and plays critical roles in embryonic development and shear stress‐mediated mechanotransduction. Ultrasound‐mediated activation of Piezo1 induces substantial Ca^2+^ influx, leading to microtubule disruption and calcium‐dependent apoptosis. Recent studies have shown that Piezo1‐mediated Ca^2+^ influx can be triggered by mechanical perturbations generated during ultrasound‐targeted MB cavitation (Figure 7b).^[56]^ This process induces mitochondrial dysfunction and promotes apoptosis in pancreatic cancer cells. In addition, the mechanosensitive channel of the large conductance (MscL) protein has been proposed as a candidate actuator for advanced sonogenetic applications. This channel responds to acoustically induced membrane strain through large conformational changes that open its transmembrane pores. Through rational protein engineering, the MscL‐I92 L mutant was developed to have a lower gating threshold (0.25 MPa) than that of the wild‐type channel. Using cationic nanoliposomes as delivery vehicles, researchers successfully achieved in vivo transfection of tumor tissues with MscL‐I92 L plasmids.^[57]^ Subsequent ultrasound stimulation triggers pronounced Ca^2+^ influx, ultimately resulting in widespread tumor cell apoptosis (Figure 7c).

To broaden the applications of sonogenetics, an ultrasonic‐to‐bioluminescent transduction platform has been engineered that utilizes in situ ultrasonic‐to‐bioluminescent transduction to enhance sono‐photodynamic therapy (SPDT).^[58]^ Upon ultrasound stimulation, mechanical gating of the MscL induces intracellular Ca^2+^ influx, which functions as a biochemical trigger for activation of the calcium‐binding photoprotein aequorin (Figure 7d). The resulting bioluminescence serves as an internal excitation source for the sonosensitizer Ce6, facilitating synergistic sono‐ and photo‐dynamic effects. Overall, these results emphasize the promise of sonogenetics as a targeted, non‐invasive approach for cancer treatment that depends on mechanically triggered biochemical signaling.

Sonogenetics combines ultrasound‐triggered calcium signaling with synthetic gene circuits to create a programmable system for immunomodulation. The influx of Ca^2+^ induced by ultrasound activates pathways such as the calcineurin‐NFAT axis, thereby enabling precise control over cytokine release, therapeutic gene activity, and the functions of adoptive immune cells. This strategy allows immune responses to be activated on demand within the tumor microenvironment while minimizing systemic immune‐related toxicity.^[60]^ However, significant translational barriers must be addressed before sonogenetics can progress toward clinical implementation. Current studies primarily rely on viral vectors and proof‐of‐concept animal models, while long‐term biosafety, gene expression stability, and off‐target activation remain insufficiently understood.[^36^, ^61^] Future efforts should focus on developing highly sensitive ultrasound‐responsive proteins, non‐viral gene delivery platforms, and programmable immune‐cell engineering strategies to facilitate clinical application, particularly for solid tumors.

#### 4.2.2 Sonomechanical therapy

**FIGURE 8:**
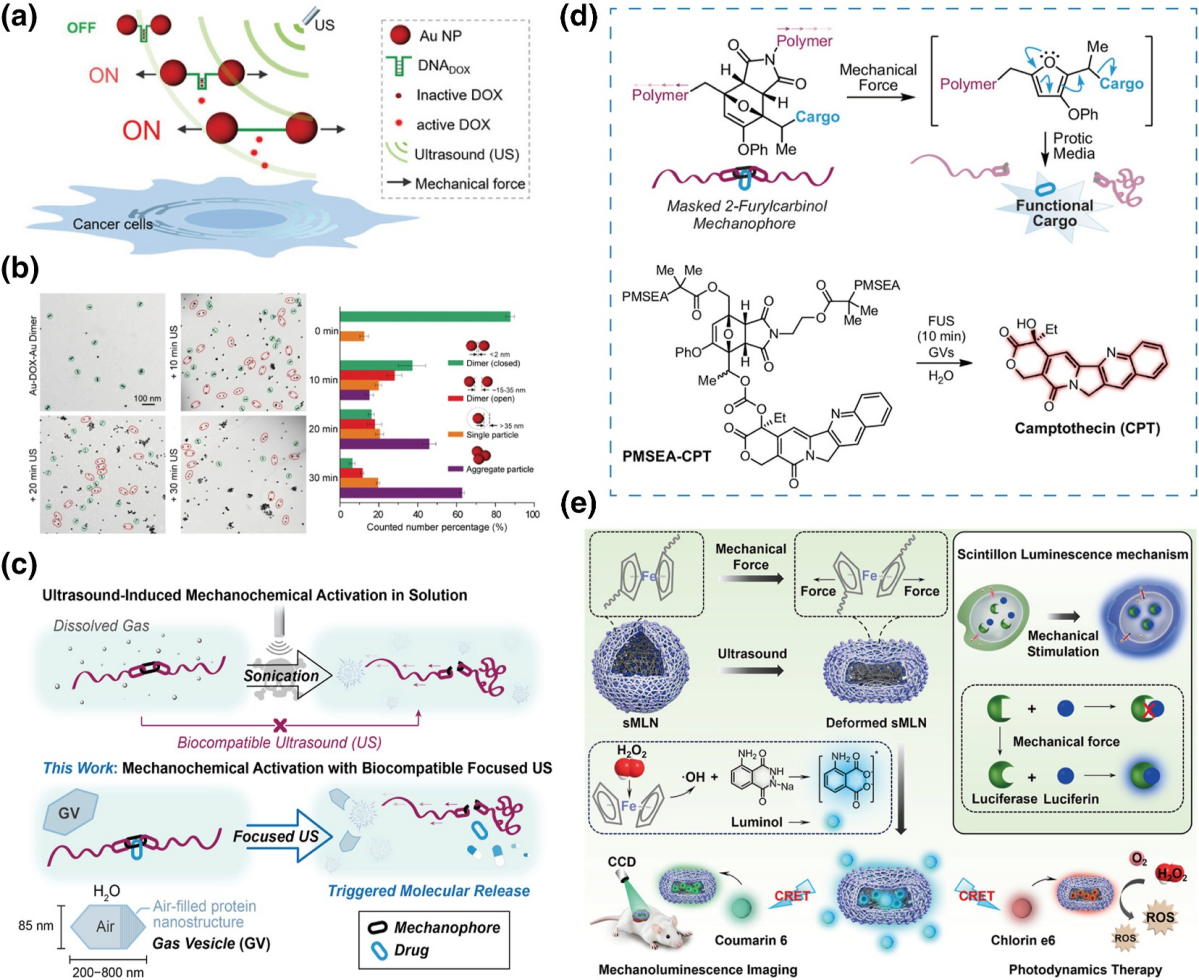
Ultrasound‐mediated mechanochemical cancer therapy. (a) Schematic illustration of the Au‐DNA dimer nanoswitch inhibiting cancer cells by ultrasound‐controlled activation of DOX. (b) TEM images of Au‐DOX‐Au dimer nanoswitches treated with different ultrasonic durations (0, 10, 20, and 30 min; green circled: closed; red circled: open). Reproduced with permission.^[62]^ Copyright 2022, The Authors. Advanced Science published by Wiley‐VCH GmbH. (c) Activation of mechanochemical reactions under physiological conditions using biocompatible FUS enabled by GVs as mechanochemical transducers. (d) Biocompatible FUS triggers release of the camptothecin from PMSEA‐CPT selectively in the presence of GVs. Reproduced with permission.^[63]^ Copyright 2023, the Author(s), Published by PNAS. (e) Schematic illustration of scintillon‐mimicking mechanoluminescence nanoreactor (sMLN) and application. Reproduced with permission.^[64]^ Copyright 2026, American Chemical Society. GVs, gas vesicles.

Driven by advances in ultrasonic mechanochemistry, sonomechanical therapy (SMT) offers strategies for drug activity. Ultrasound‐induced bond scission can release therapeutics from inactive parent molecules.^[65]^ One example is the mechano‐responsive nanoswitch developed by Herrmann and colleagues. Under ultrasound irradiation, the nanoswitch undergoes a conformational transition that disrupts non‐covalent interactions and releases doxorubicin (DOX), inducing DNA damage in tumor cells (Figure 8a).^[62]^ Although this platform enables mechanically activated tumor suppression (Figure 8b), the 20 kHz irradiation used for these mechanochemical processes is generally unsuitable for clinical application.

To couple biocompatible ultrasound with covalent mechanochemistry, Robb and colleagues used proteinaceous GVs as acoustomechanical transducers.^[63]^ Under physiological FUS conditions, these GVs facilitate mechanochemical activation of mechanophore‐functionalized polymers and controlled payload release (Figure 8c). Camptothecin (CPT) was conjugated to a mechanophore bis‐initiator via a carbonate linkage. GVs‐mediated FUS exposure for 10 min released approximately 8% of the CPT and significantly reduced cell viability.

Building upon these advances in ultrasound‐triggered mechanochemistry, mechanoluminescent systems have also been developed to enhance PDT. In a representative study, scintillon‐mimetic mechanoluminescent nanoreactors (sMLNs) have been designed to convert ultrasonic energy into localized luminescence (Figure 8d).^[64]^ Under ultrasound irradiation, acoustic shear forces induce conformational deformation of the ferrocene moiety via stretching and twisting. This distortion enhances electron density at the Fe center and reduces sterics, favouring substrate binding. The alterations trigger a Fenton‐like reaction, leading to the formation of luminol‐based luminescence that acts as a PDT internal excitation source.

Apart from the physical damage to the tumor, mechanical stimulation may activate signalling cascades within cells and promote changes in their cytoskeletons, triggering stress‐related cell death pathways and enhancing release of immune‐potentiating molecules. In addition, localized mechanical disruption may soften the extracellular matrix and facilitate transport, thereby improving the infiltration of immune cells into the tumor, making SMT a promising mechanical priming strategy for combination cancer immunotherapy. Many researchers have recently become interested in mechanotransduction and sonodynamic approaches, which utilize ultrasound. However, this area is still early in its development. Thus, it is not yet fully clear the exact relationships between ultrasound parameters, force transmittance, mechanophore activation, and therapeutic efficacy. Future studies should focus on mechanochemical systems that work in a more sensitive way so that there can be a quantitative framework put in place for designing US‐responsive mechanophores. Also, the therapeutic efficacy and safety of these US‐responsive mechanophores should be validated in tumor models.

### 4.3 US‐activated physicochemical therapies

US‐activated physicochemical therapies primarily use ROS as their cytotoxic effect, but they produce these species via different material‐specific processes. Both SDT and SPT convert ultrasound energy into localized signals that induce ROS, thereby causing tumor‐cell damage. In SDT, acoustic cavitation activates sonosensitizers through sonochemical reactions, producing ROS. It has strong preclinical backing and is currently in clinical trials. Conversely, SPT has recently progressed by utilizing piezoelectric nanomaterials. Unlike SDT, SPT creates ROS through piezoelectric charge separation without depending on traditional sonosensitizers.

#### 4.3.1 Sonodynamic therapy (SDT)

SDT is an emerging therapeutic modality that combines low‐intensity ultrasound with sonosensitizers to induce cytotoxic effects through acoustic cavitation within target tissues. A bibliometric analysis of the Web of Science database using the keyword “sonodynamic therapy” identified three distinct phases in the development of SDT research: an initial foundational stage (1989–2006), a period of steady growth (2007–2016), and a phase of rapid expansion beginning in 2017.^[66]^ Given the increasing global demand for innovative cancer therapies, research activity in this field is expected to continue expanding in the coming years.[^67^, ^68^]

The development of integrated SDT systems has become a key focus in oncological engineering. The efficacy of these platforms depends on precise spatiotemporal control of ultrasound delivery, rigorous calibration of acoustic parameters, and accurate monitoring of localized ROS generation and temperature changes.^[69]^ Despite the increasing number of in vitro stimulation methods, substantial variability across experimental setups often leads to inconsistent cellular responses, even under comparable acoustic conditions.[^70^, ^71^, ^72^] This lack of methodological standardization remains a major challenge because it limits the reliable translation of in vitro sonodynamic findings into predictable in vivo therapeutic outcomes.

**FIGURE 9:**
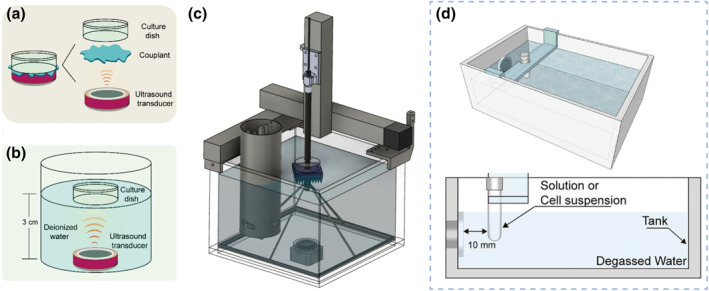
In vitro experimental configurations for SDT. (a) Sonodynamic treatment using couplant as ultrasonic medium. (b) Sonodynamic treatment using deionized water as ultrasonic medium. Reproduced with permission.^[73]^ Copyright 2023, Springer Nature. (c) Water tank setup with absorbing structures located above the bottom‐mounted ultrasonic transducer. Reproduced under terms of the CC‐BY license.^[74]^ Copyright 2025, The Author(s), published by De Gruyter. (d) A three‐dimensional model and a side view of a free‐field ultrasound system. Reproduced with permission.^[75]^ Copyright 2025, Wiley‐VCH.

Most current in vitro systems operate under standing‐wave conditions generated by reflection of ultrasound at the air‐liquid interface of the culture medium (Figure 9a,b). Under these circumstances, adherent cells are exposed to spatially nonuniform acoustic fields, making the effective acoustic dose difficult to define with precision. Attempts to suppress wave reflection commonly involve placing acoustic absorbers above the medium surface (Figure 9c); however, these configurations often require partial opening of the culture system and therefore introduce an increased risk of contamination.^[76]^ More importantly, they do not fully resolve the problem of acoustic instability within the exposure chamber.

To address these limitations, a free‐field ultrasound platform has been developed to deliver more controlled and reproducible acoustic stimulation.^[77]^ Similar free‐field configurations reported in the literature have also demonstrated improved acoustic uniformity and reduced standing‐wave artifacts.[^78^, ^79^] The resulting system provides stable acoustic pressure output and can be applied across both cellular and animal models (Figure 9d).^[75]^ Unlike conventional standing‐wave setups, the free‐field configuration minimizes acoustic interference and substantially reduces unintended mechanical or thermal injury unrelated to sonosensitizer activation. This distinction is particularly important in SDT studies, where nonspecific ultrasound‐induced damage can easily obscure the true contribution of the sonodynamic process. Under free‐field conditions, the observed therapeutic responses are therefore more likely to reflect genuine interactions between ultrasound and the sensitizing agents. The improved acoustic controllability also facilitates mechanistic studies of ultrasound‐mediated tumor cell responses and enables a more reliable optimization of SDT parameters. Based on this platform, we further established a standardized workflow for sonosensitizer activation, extending from in vitro assays to in vivo therapeutic evaluation. Together, the free‐field exposure system and accompanying protocol provide a reproducible experimental framework that should improve interlaboratory consistency and support the continued translational development of SDT.

Successful clinical translation of SDT depends not only on advances in ultrasound delivery technologies but also on the development of sonosensitizers with strong acoustic responsiveness and favorable biosafety profiles. To meet these requirements, a broad range of materials has been explored, including conventional organic molecules and multifunctional nanostructured systems, each possessing distinct pharmacological and therapeutic characteristics.[^80^, ^81^] Among these candidates, organic small‐molecule sonosensitizers have attracted particular attention because their chemical structures, physicochemical properties, and metabolic behaviors are generally well characterized, thereby providing advantages for mechanistic investigation and translational evaluation.[^38^, ^82^] Based on their structural features, currently reported organic sonosensitizers can be broadly divided into the following categories: porphyrins,^[83]^ phthalocyanines,^[84]^ xanthene derivatives,^[85]^ metal complexes,[^86^, ^87^, ^88^] natural products,^[89]^ indocyanines, and others (Table 2). Notably, many of these sonosensitizers were originally developed as photosensitizers for photodynamic therapy (PDT) or were derived from established photosensitising scaffolds through structural modification.^[107]^ This close relationship between PDT and SDT agents offers an important translational advantage, as clinically approved photosensitizers already have substantial safety and pharmacokinetic data, which may accelerate their regulatory adaptation for SDT applications. However, efficient responsiveness to light irradiation does not necessarily confer effective ultrasound activation. Consequently, many existing photosensitizers exhibit limited sonodynamic efficiency under clinically relevant acoustic conditions, thereby restricting their therapeutic performance. These limitations have driven increasing interest in the rational engineering of photosensitizer structures to enhance ultrasound responsiveness. Through these optimization strategies, next‐generation sonosensitizers may achieve improved therapeutic efficacy and greater potential for clinical translation.

**TABLE 2:** Organic small‐molecule sonosensitizers and their potential sonodynamic applications.

Category	Sonosensitizers	In vitro, in vivo, or both	Power	Bio‐model	Dosing	Cytotoxicity (%)	Refs.
Porphyrins or their derivatives	Hematoporphyrin	In vitro	1.75 MHz, 1.4 ± 0.07 W/cm^2^, 3 min	S180 cell line	20 μg/mL	60.51	^[90]^
	Protoporphyrin IX	In vitro	1 MHz, 0.16 W/cm^2^, 60 s	Rat glioma cell line C6 and human glioblastoma cell line U87 MG	1.0 μg/mL	21.1	^[91]^
	Phytochlorin	Both	1 MHz, 1.0 W/cm^2^, 60 s*; 1 MHz, 0.4–1.6 W/cm^2^, 180 s†	Human lung adenocarcinoma cell SPCA‐1; Kunming (KM) female mice	0.2 mg/mL*, 10–40 mg/mL†	74.23	^[92]^
	Npe6	Both	2 MHz, 4.5 W/cm^2^, 60 s*; 2 MHz, 3 W/cm^2^, 15 min†	Sarcoma 180 cells*; BALB/c (Colon 26 carcinoma cells)	80 μM*, 25 mg/kg†	94.7	^[92]^
	HMME	In vivo	1 MHz, 0.5 W/cm^2^, 120 s	C6 glioma animal model (Wistar rats)	10 ng/kg†	/	^[93]^
	DVDMs	In vitro	0.97 MHz, 0.52 MPa, 3 min	Human glioblastoma cell line U87 MG	10 μg/mL*	51.3	^[94]^
	C34	Both	1.0 MHz, 3.21 W/cm^2^, 10 min*; 1.0 MHz, 1.88 W/cm^2^, 30 min†	MCF‐7 human breast‐cancer cells and 4T1 breast‐cancer model	50 μM*; 16 mg/kg†	41.44	^[77]^
Phthalocyanines	AlPcTS	In vivo	1.92 MHz, 3 W/cm^2^, 15 min	BALB/c (Colon 26 carcinoma cells)	2.5 mg/kg	/	^[95]^
	ZnPcS_2_P_2_	In vitro	1.0 MHz, 0.5 W/cm^2^, 2 min	U251 glioma cells	5.0 μg/mL	>80	^[96]^
Xanthenes	RBD	In vitro	1.92 MHz, 2.3 W/cm^2^, 15 s	Sarcoma 180 cells	100 µM	>90	^[97]^
	RBD4	In vitro	1 MHz, 2 W/cm^2^, 3 min	Human hepatocellular carcinoma HepG2	0.5 μM	>70	^[98]^
Metal complexes	[Ru(bpy)_3_]^2+^	Both	3 MHz, 0.3 W/cm^2^, 20 min	4T1 tumor cells and 4T1 tumor‐bearing mice model	10 μM*; 0.5 mg/kg†	>80	^[99]^
	IrTMPPS	Both	3 MHz, 0.3 W/cm^2^, 20 min	4T1 tumor cells and 4T1 tumor‐bearing mice model	10 μM*; 25 μL, 500 μM†	>80	^[100]^
Natural products	Curcumin	In vitro	1.7 MHz, 0.46 W/cm^2^, 8 s	CNE nasopharyngeal carcinoma cells	10 μM	86.67	^[101]^
	Hypocrellin B	In vitro	0.84 MHz, 0.5 W/cm^2^, 60 s	Human gastric adenocarcinoma cell line SGC7901 and SGC7901/ADR	5 μM	58.94/78.72	^[102]^
Indocyanines	ICG	In vitro	1 MHz, 1.0 W/cm^2^, 2 min	Human fibroblast‐like synoviocyte MH7A cell line	50 μg/mL	45.76	^[103]^
	IR‐780	In vitro	1.505 MHz, 0.63 W/cm^2^, 3 min	Human pancreatic cancer cell line BxPC‐3	5 μg/mL	42.79	^[104]^
Others	MB6C	In vitro	1.7 MHz, 0.5 W/cm^2^, 20 min	A549 lung cancer cells	20 μM	>90	^[105]^
	SPFX	In vitro	2 MHz, 2 W/cm^2^, 30s	Sarcoma 180 cells	50 μM	55.85	^[106]^

Abbreviations: *for in vitro models; ‐, means not disclosure; /, means not involve; †, for in vivo models; AlPcTS, chloroaluminum phthalocyanine tetrasulfonate; DVDMs, sinoporphyrin sodium; FF, free‐field; HMME, hematoporphyrin monomethyl ether; ICG, Indocyanine green; IR780, 2‐[2‐[2‐Chloro‐3‐[(1,3‐dihydro‐3,3‐dimethyl‐1‐propyl‐2H‐indol‐2‐ylidene)ethylidene]‐1‐cyclohexen‐1‐yl]ethenyl]‐3,3‐dimethyl‐1‐propylindolium iodide; IrTMPPS, iridium(III)‐porphyrin sonosensitizer; MB6C, 3,7‐bis(dihexylamino)‐phenothiazin‐5‐ium iodide; Npe6, mono‐l‐aspartyl chlorin e6; RBD, rose bengal derivatives; SPFX, sparfloxacin.SW, standing wave.

**FIGURE 10:**
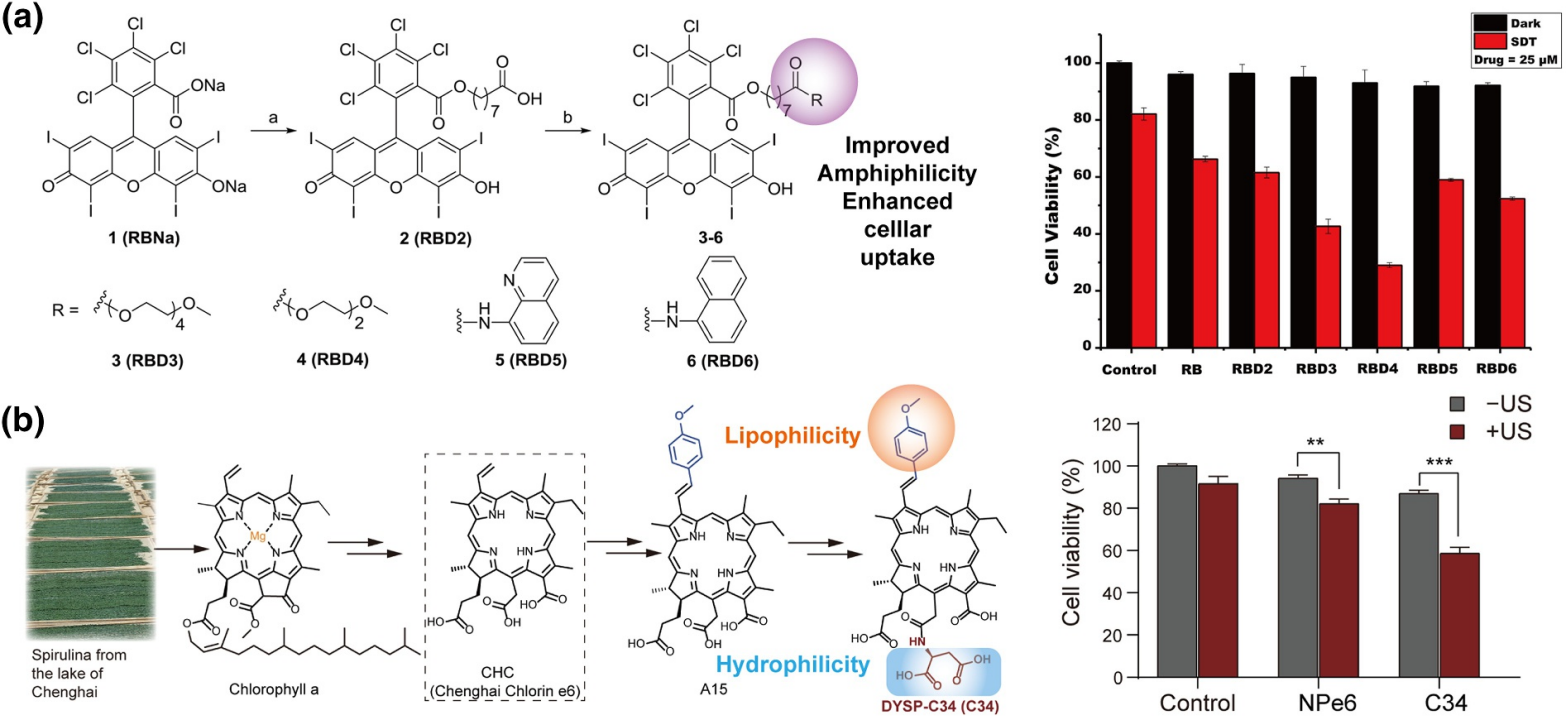
Amphiphilic modification strategy for organic sonosensitizers. (a) Chemical structure and sonodynamic anticancer effects of RBDs. Reproduced with permission.^[98]^ Copyright 2018, Elsevier. (b) Schematic showing the preparation process of C34 and the sonotoxicity of C34 and NPe6 against MCF‐7 cells. Reproduced with permission.^[77]^ Copyright 2021, American Association for the Advancement of Science.

Among the various molecular optimization strategies, amphiphilic design has emerged as a promising approach because it can simultaneously enhance ultrasonic sensitization and biological delivery.^[108]^ As exemplified by Chen et al., the integration of mPEG and amine‐based groups into Rose Bengal (Figure 10a) yields derivatives with optimized hydrophilic‐lipophilic balance, thereby markedly enhancing ROS generation and therapeutic efficacy.^[98]^ Building on this strategy, C34 was developed as an ultrasound‐responsive “molecular machine” (Figure 10b).^[77]^ Through precise regioselective substitution, C34 achieves an optimized amphiphilic balance that not only promotes preferential tumor accumulation, but also enhances ultrasound‐triggered cytotoxicity. The amphiphilic architecture reduces interfacial surface tension and facilitates the preferential enrichment of the sonosensitizer at cavitation nuclei and gas‐liquid interfaces. In parallel, the amphiphilic framework also contributes to improved cellular uptake and intracellular bioavailability, further strengthening therapeutic performance. Collectively, these findings demonstrate that rational amphiphilic modification can simultaneously optimize ultrasonic responsiveness and biological delivery, highlighting its potential as an effective strategy for developing next‐generation sonosensitizers with enhanced therapeutic efficacy and translational potential.

Recent advances in materials engineering have expanded the range of sonosensitizers to include various inorganic nanomaterials that provide special physicochemical characteristics.[^109^, ^110^] Titanium dioxide (TiO_2_) nanoparticles are one of the most preferred inorganic sonosensitizers. Ultrasound application causes cavitation‐driven sonochemical activation, resulting in electron‐hole pair generation on the surface of TiO_2_, which leads to ROS formation. Defect engineering, adjuvant of noble metals like Au or Pt, or heteroatom doping of TiO_2_ (or other sonocatalyst) can improve sonodynamic effects by narrowing the bandgap and enhancing charge separation. Furthermore, various carbon‐based nanomaterials, such as layered graphene and reduced graphene oxide, exhibited great potential and promise on account of their large specific surface areas, numerous catalytic active sites, and efficient interfacial charge transfer, all of which boost US‐induced ROS production.[^68^, ^111^] Recently, metal‐organic frameworks and various hybrid inorganic nanostructures gained significant interest and versatility as sonosensitizers. Owing to their customizable compositions, porous structures, and catalytic metal ions, they facilitate oxygen production, ROS generation, and therapeutic agent loading. Therefore, they act as multifunctional platforms for combination therapy.[^112^, ^113^]

Organic and inorganic sonosensitizers each have unique benefits but also face similar challenges, such as poor water solubility, rapid clearance from the body, limited accumulation in tumors, and low selectivity. Thus, enhancing their delivery to tumors is just as crucial as improving their inherent sensitizing abilities. Ideally, these agents should accumulate efficiently in tumors while minimizing toxicity to healthy tissues. Researchers have addressed these constraints by encapsulating sonosensitizers in micro‐ or nanocarriers and by designing self‐assembled nanosystems.^[114]^ The rapid growth of SDT research since 2017 has coincided with the convergence of nanotechnology and ultrasound medicine.^[115]^ Continued advances in molecular and materials design may further expand this field.

**FIGURE 11:**
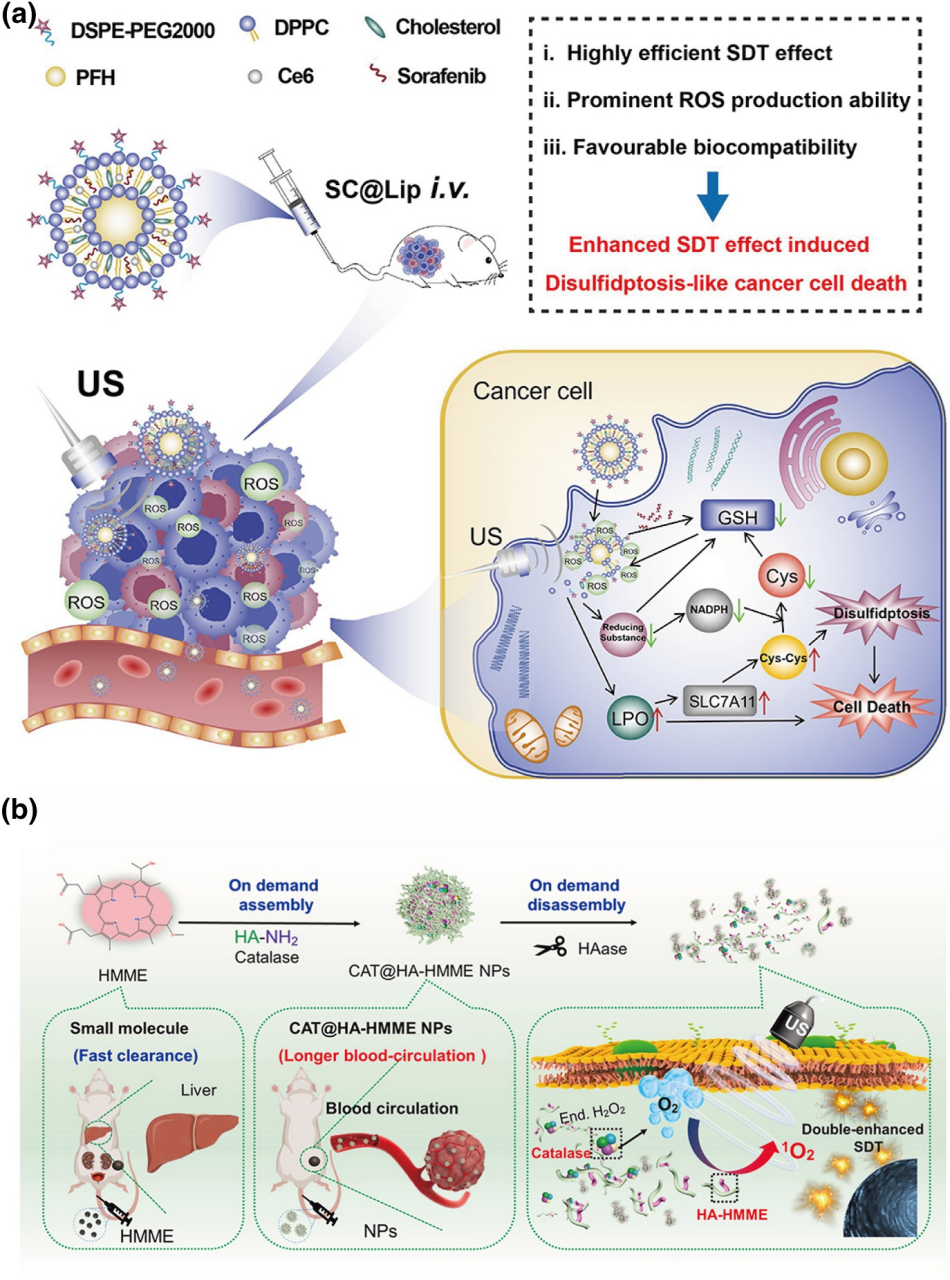
Design and application of nanoparticles for sonodynamic cancer therapy. (a) Schematic of SDT with SC@Lip liposomes. Reproduced with permission.^[116]^ Copyright 2024, Dove Medical Press Ltd. (b) Schematic of SDT with CAT@HA‐HMME nanoparticles. Reproduced with permission.^[117]^ Copyright 2022, Keai Publishing Ltd.

Nanostructural strategies, including liposomal or polymeric encapsulation, biomimetic carriers, porous‐material loading, and nanoparticle‐surface conjugation, can improve sonosensitizer solubility and circulation time, thereby strengthening the sonodynamic response.^[118]^ Li and colleagues developed PEG‐modified liposomes co‐loaded with sorafenib and Ce6 (SC@Lip) by rotary evaporation (Figure 11a).^[116]^ Under US irradiation, SC@Lip generated ROS and promoted intracellular lipid‐peroxide accumulation. After uptake by 4T1 cells, SC@Lip‐mediated SDT reduced cell viability to below 5%. In a 4T1 subcutaneous xenograft model, this treatment significantly inhibited tumor growth and prolonged survival, supporting nanoliposomes as sonosensitizer‐delivery vehicles. To address limitations associated with the tumor microenvironment (TME), Chen and colleagues further engineered self‐assembled hyaluronic acid nanoparticles (CAT@HA‐HMME) that co‐encapsulated hematoporphyrin monomethyl ether ,HMME and catalase (CAT).^[116]^ After receptor‐mediated endocytosis, hyaluronidase in the TME triggered nanoparticle disassembly (Figure 11b). Released CAT converted endogenous H_2_O_2_ into O_2_, alleviating tumor hypoxia and enhancing HMME‐mediated ROS generation. Pharmacokinetic analysis showed that the nanoparticles prolonged the blood‐circulation half‐life (t_1/2_ = 4.2 h) relative to free HMME (t_1/2_ = 0.8 h) and exhibited favorable biosafety. Together, these effects enhanced oxidative damage and tumor suppression.

While SDT nanoplatforms have demonstrated encouraging preclinical efficacy, their clinical translation remains constrained by several challenges, including limited ROS‐generating efficiency, insufficient tumor accumulation, tumor hypoxia, biosafety concerns, and a lack of standardized ultrasound dosimetry. To overcome these limitations, sonosensitizers and their nanocarriers need to be designed with reproducible degradation, well‐tolerated and optimized pharmacokinetics. The intent of the above strategy is to enhance therapeutic activity while lowering the systemic toxicity. Nanoplatforms with multifunctionality not only facilitate drug delivery but also serve as an effective platform for sono‐immunomodulation when combined with immunotherapeutics such as immune checkpoints and immune adjuvants. The use of this combination enhances ROS‐mediated immunogenic cell death, boosts antigen‐specific immune responses, and increases systemic antitumor ability. Efforts going forward should include the establishment of highly efficient clinical scalable sonosensitizers, standardized ultrasound exposures and acoustic dosimetries, and multifunctional nanoplatforms that elevate therapeutic indices, biosafety, and immunomodulation. These procedures will facilitate the clinical translation of SDT.

#### 4.3.2 Sonopiezoelectric therapy (SPT)

SPT causes the generation of ROS using charge separation due to the piezoelectric effect as opposed to conventional sonosensitizers.^[119]^ This new approach relies on the use of piezoelectric materials, which generate charge on the surface under mechanical stress. Moreover, the surface charge that forms due to the mechanical stress is proportional to the applied acoustic pressure. These materials are sonosensitizers whose ultrasound irradiation can generate charge separation and induce the formation of electron‐hole (e^−^‐h^+^) pairs and an internal electric field.^[120]^ The subsequent migration of charge carriers to the material‐liquid interface enables redox reactions and the generation of ROS, facilitating localized tumor ablation.^[121]^

Besides cell killing via ROS, SPT‐generated transient electric fields have a direct effect on tumor microenvironment. Through non‐chemical means, these low‐power electric signals can change the electrical behaviour of cancer cells to slow cell growth. It is noteworthy that electrical stimulation may lower or circumvent multidrug resistance (MDR), thereby potentially enhancing the sensitivity of resistant tumor cells. Localized electrical stimulation also triggers antigen release and enhances dendritic cell activation, highlighting its growing immunomodulatory potential.[^124^, ^125^] Combining oxidative stress induction with direct electrobiological modulation through piezoelectric‐based SPT creates a versatile platform to deliver advanced non‐invasive cancer treatments and to explore new combinations of immunotherapy.

**FIGURE 12:**
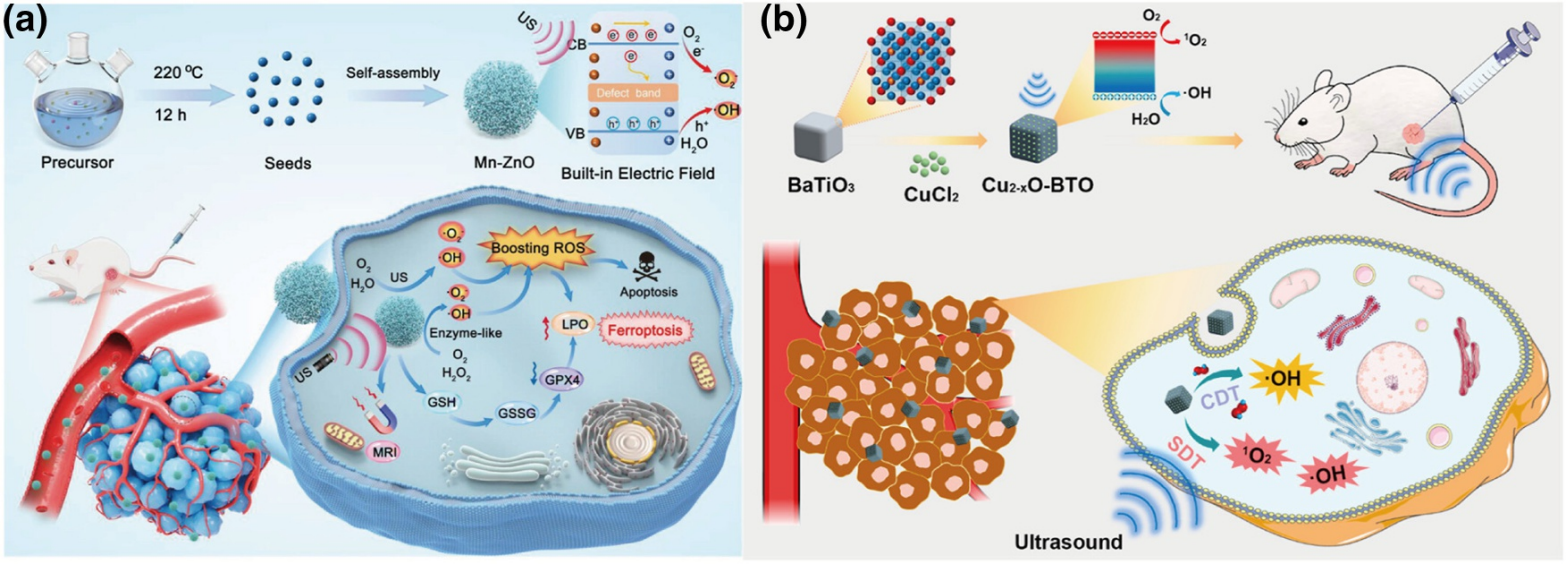
Piezoelectric nanoplatforms for SPT. (a) Mesoporous Mn‐ZnO nanocluster and its anticancer mechanism of enhanced piezocatalytic therapy and ferroptosis. Reproduced with permission.^[122]^ Copyright 2023, Wiley‐VCH. (b) Enhanced sonodynamic and chemodynamic therapy based on Cu_2‐x_O‐BTO NCs. Reproduced with permission.^[123]^ Copyright 2022, American Chemical Society.

As a representative piezoelectric material, Zinc Oxide (ZnO) has been widely used in ultrasonic transducers since the late 1990s. Despite its established utility, the relatively low intrinsic piezoelectric efficiency of ZnO can limit its effectiveness in ROS‐mediated sonocatalysis. To overcome this limitation, recent studies have explored metal‐ion doping strategies to enhance the catalytic activity of ZnO nanoparticles.^[122]^ For instance, Tian et al. incorporated Mn dopants into the ZnO lattice (Figure 12a), inducing structural modifications that significantly enhanced internal polarization. This lattice engineering strategy not only improved the piezoelectric response but also suppressed the recombination of e^−^‐h^+^ pairs at oxygen‐vacancy sites. Consequently, the modified ZnO nanoparticles exhibited enhanced ROS generation under ultrasound stimulation, highlighting their potential for sonopiezoelectric therapeutic applications.

Another important candidate for SPT is Barium Titanate (BaTiO_3_), a ferroelectric perovskite well known for its strong piezoelectric properties since the 1970s. Early studies by Zhu et al. demonstrated that pure BaTiO_3_ nanoparticles possess intrinsic ultrasound‐sensitizing activity. Under ultrasound stimulation, these nanoparticles generate ROS that induce tumor cell death. To further improve the catalytic performance, recent studies have focused on constructing piezoelectric heterostructures. For instance, Zhao et al. developed Cu_2‐x_O‐BaTiO_3_ composite nanoparticles through a facile two‐step synthesis process (Figure 12b).^[123]^ In this composite system, BaTiO_3_ functions as the primary piezoelectric scaffold. Upon ultrasound stimulation, the generated e^−^‐h^+^ pairs are efficiently separated. Owing to favorable band alignment, these charge carriers preferentially migrate toward the Cu_2‐x_O component, which possesses lower energy states. Continuous ultrasound exposure maintains a potential difference between the material energy levels and the redox potentials of the surrounding medium. This directed charge transfer enhances ROS generation, highlighting the potential of heterojunction engineering for improving sonopiezoelectric performance. Despite substantial advances, all classes of piezoelectric materials still face inherent limitations, including insufficient piezoelectric efficiency, limited biocompatibility, and inadequate long‐term stability. Consequently, future research should prioritize systematic optimization of these materials. Ultimately, balancing high catalytic activity with favorable biocompatibility is critical for the development of next‐generation SPTs.

### 4.4 Sono‐immunomodulation

Cancer immunotherapy is limited by variable response rates and immune‐related adverse effects. Sono‐immunotherapeutic approaches use biomedical ultrasound to enhance local treatment and antitumor immunity. By tuning acoustic parameters, non‐thermal ultrasound can influence several stages of the cancer‐immunity cycle and may improve responses to immunotherapy.[^126^, ^127^] The preceding sections show that non‐thermal US modalities elicit antitumor immunity through distinct mechanisms. Here, we identify the shared immunomodulatory principles underlying these responses. Despite their different initiating mechanisms, these modalities can remodel the tumor immune microenvironment and converge on the cancer‐immunity cycle. Therefore, nonthermal US may provide a versatile platform for spatially and temporally controlled cancer immunomodulation.

**FIGURE 13:**
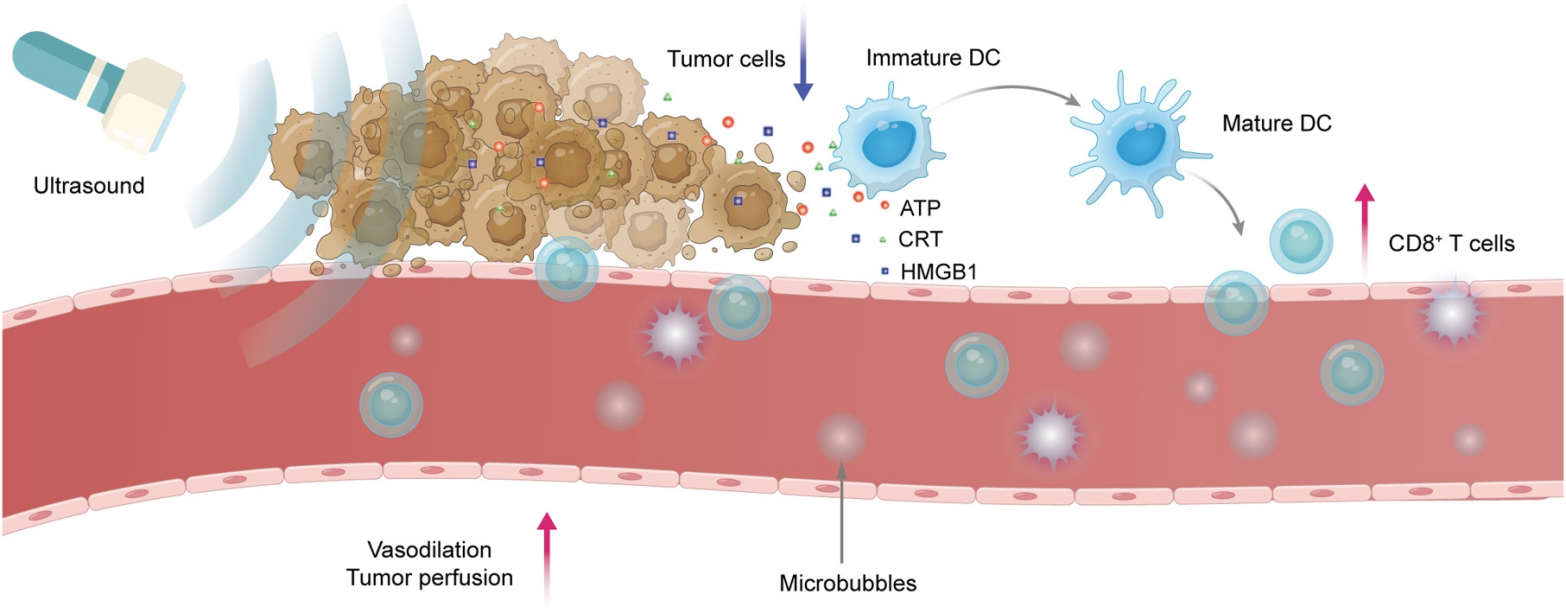
Mechanisms of non‐thermal ultrasound‐enhanced sono‐immunotherapy.

Non‐thermal US can increase endothelial and cellular permeability, remodel extracellular‐matrix barriers, and improve local perfusion. Cavitation and mechanical forces may also increase the permeability of abnormal tumor vasculature, facilitating immune‐cell infiltration and intratumoral delivery of immunomodulators.^[128]^ Therefore, nonthermal US should be considered as both a local physical stimulus and a spatiotemporally controllable regulator of the tumor immune microenvironment. It may reinforce immune priming by inducing immunogenic stress and promoting the release or exposure of TAAs and DAMPs. These signals contribute to DC maturation, antigen processing and cross‐presentation, and subsequent activation of CD8^+^ CTLs (Figure 13). Together, these mechanisms provide a basis for integrating non‐thermal ultrasound with cancer immunotherapy.^[129]^

With the integration of immune adjuvants into vaccines, the development of ultrasound‐activated in situ vaccines represents a promising direction. For example, the R837‐loaded liposome PEOz‐Lip@R837 cancer vaccine generated TAAs under ultrasound cavitation in pancreatic cancer‐bearing mice.^[130]^ TAAs and R837 collectively promoted DC maturation and downregulated multiple immune checkpoint molecules, including CD274, Foxp3, and CTLA4, in Panc02 tumors, which further enhanced the activation of CD8^+^ T cells. These properties make sono‐immunotherapy particularly attractive as a combinational strategy. Ultrasound‐responsive microbubbles, nanobubbles, and sonosensitizer‐based nanosystems can be engineered to co‐deliver immune adjuvants, checkpoint inhibitors, cytokines, or nucleic‐acid therapeutics and release them on demand at the tumor site, thereby enhancing local efficacy while reducing systemic toxicity. Apart from relying on the performance of immunomodulators, the immune effects induced by biomaterial components have gained increased attention in recent years. Inorganic materials shine in this area because they can release immunostimulating metal ions. For instance, Mn‐based phenolic nanoadjuvants were fabricated to induce a combination of SDT‐mediated ICD and Mn^2+^‐promoted activation of the cGAS‐STING pathway.^[131]^ These effects significantly increased the percentage of CD86^+^ DCs in TDLNs from 4T1 tumor‐bearing mice compared with controls. Other bioactive metal‐based biomaterials, such as magnesium‐, calcium‐, molybdenum‐, and zinc‐based materials, have been shown to be effective for “sono‐metalloimmunotherapy” in certain cancer‐ridden mouse models.^[132]^ In summary, ultrasound can induce ICD in cancer cells through non‐thermal effects, which in turn initiate innate and adaptive immune responses to some extent. Upon ultrasound exposure, blood vessels and cells can become more permeable, creating favorable conditions that may enhance the effectiveness of certain combination cancer treatments.

## 5 CONCLUSIONS AND PERSPECTIVES

Modern oncology is witnessing a shift towards precision medicine and an integrated multidisciplinary approach. Within this context, non‐thermal US is a versatile toolkit to spatially control cancer therapy by linking acoustic energy to mechanical, cavitation‐mediated and physicochemical bioeffects. Due to its non‐invasive delivery and effective penetration depth, it has a variety of applications, including tissue fractionation, drug delivery, mechanotransduction, and ROS generation. Interdisciplinary technologies will continue to gain importance, especially AI‐assisted imaging and advanced nanomaterials.

Therapeutic modalities remain hampered by their own translational challenges. Histotripsy is one of the most clinically advanced treatments and requires specialized and sophisticated instrumentation, imaging guidance, and exact patient selection to avoid damage to adjacent structures. A limitation of SDT is the tumor‐specificity of current sonosensitizers and the dearth of standardized acoustic dosimetry, making study comparisons and regulatory approval assessments complicated. The transient effect of cavitation‐enhanced permeability and the short circulation time of typical microbubbles are the issues to be tackled by UTMD. Sonogenetics struggles with efficiently delivering genes and maintaining prolonged transgene expression. SMT and SPT, being more recently developed, require extensive validation of their safety, pharmacokinetics, and in vivo efficacy. Overcoming these modality‐specific limitations will require coordinated progress across materials science, acoustic engineering, molecular biology, and clinical trial design to accelerate clinical translation.

Beyond modality‐specific challenges, several broader issues must be resolved before non‐thermal US can be widely adopted in clinical oncology:

1. Sono‐therapy involves both theoretical and practical risks. A primary concern is that acoustic mechanical forces might dislodge surviving cancer cells into the blood vessel and lead to systemic metastasis. Under certain exposure circumstances, local vascular problems can emerge, such as an injury to the endothelium and thrombosis. Although non‐thermal US is non‐invasive, it is possibly not safe in the long term. As such, stringent and standard safety criteria need to be developed for its use.
2. Ultrasound‐based cancer therapies are still in preclinical development, with few in the clinical phase. Major obstacles comprise the lack of standardized instrumentation and the absence of standard dosimetric protocols. There are different constraints based on the delivery mode. FUS is dependent on integrating advanced image‐guidance technologies such as MRI or diagnostic US, making it technically complex and costly for routine laboratory research. Although the Non‐FUS is inexpensive and simpler and allows for even exposure of larger tumor volumes, it has less spatial control. The clinical value of focused versus non‐focused systems will depend on a number of factors including indication, target anatomy, and endpoint. In preclinical research, the operational simplicity of non‐FUS may facilitate broader deployment and higher‐throughput studies.
3. Precise acoustic dosimetry remains difficult because sound‐tissue interactions vary in vivo. Tissue heterogeneity, physiological motion, and physical interfaces can affect the local acoustic field, so the in situ dose can be quite different from the nominal transducer output. Predicting energy deposition at depth is complicated by wavefront distortion and harmonic generation when therapeutic ultrasound operates in a nonlinear regime. Such uncertainty can generate inadvertent damage to tissues. For reliable and safe sono‐therapies, it is essential to account for these uncertainties, highlighting the importance of standardized reporting and real‐time monitoring.

## CONFLICT OF INTEREST STATEMENT

Mingle Li is a young editorial board member of *Smart Molecules* and co‐author of this article. They were excluded from editorial decision‐making related to the acceptance of this article for publication in the journal.

## Data Availability

Data sharing not applicable to this article as no datasets were generated or analysed during the current study.
